# Development of a Cationic Amphiphilic Helical Peptidomimetic (B18L) As A Novel Anti-Cancer Drug Lead

**DOI:** 10.3390/cancers12092448

**Published:** 2020-08-28

**Authors:** Yuan Lyu, Steven Kopcho, Folnetti A. Alvarez, Bryson C. Okeoma, Chioma M. Okeoma

**Affiliations:** 1 Department of Pharmacology, Stony Brook University Renaissance School of Medicine, Stony Brook, NY 11794, USA; yuan.lyu@stonybrook.edu (Y.L.); steven.kopcho@stonybrook.edu (S.K.); folnetti.alvarez@stonybrook.edu (F.A.A.); 2 Department of Psychiatry, Stony Brook University Hospital, Stony Brook, NY 11794, USA; bryson.okeoma@stonybrookmedicine.edu

**Keywords:** anti-cancer peptide, B18L, BST-2, drug resistance, breast cancer cells

## Abstract

**Simple Summary:**

We have previously shown that overexpression of BST-2 in breast cancer cells promotes cancer cell adhesion and aggressiveness. Although the level of BST-2 in breast tumors is higher than those of established cancer markers, there is no therapy targeting BST-2 in cancer cells. Thus, we developed the first-generation BST-2-based peptide series―B49/B49Mod1 that impairs adhesion-dependent biological events in breast cancer cells and inhibits tumor growth. Using sequence/structure modification and bioactivity guided separation, we identified B18 as the minimal sequence required for the anti-adhesion activity of B49Mod1. The current study demonstrates that a derivative of B18, a cationic amphiphilic α-helical peptidomimetic B18L, kills drug-resistant and drug-sensitive breast cancer cells. B18L impairs cancer cell membrane and dysregulates mitochondrial and signaling events necessary for the survival of cancer cells. This study provides the first evidence that a BST-2-based peptide (B18L) is a promising therapeutic agent for treatment of breast cancers, thus supporting further development.

**Abstract:**

BST-2 is a novel driver of cancer progression whose expression confers oncogenic properties to breast cancer cells. As such, targeting BST-2 in tumors may be an effective therapeutic approach against breast cancer. Here, we sought to develop potent cytotoxic anti-cancer agent using the second-generation BST-2-based anti-adhesion peptide, B18, as backbone. To this end, we designed a series of five B18-derived peptidomimetics. Among these, B18L, a cationic amphiphilic α-helical peptidomimetic, was selected as the drug lead because it displayed superior anti-cancer activity against both drug-resistant and drug-sensitive cancer cells, with minimal toxicity on normal cells. Probing mechanism of action using molecular dynamics simulations, biochemical and membrane biophysics studies, we observed that B18L binds BST-2 and possesses membranolytic characteristics. Furthermore, molecular biology studies show that B18L dysregulates cancer signaling pathways resulting in decreased Src and Erk1/2 phosphorylation, increased expression of pro-apoptotic Bcl2 proteins, caspase 3 cleavage products, as well as processing of the caspase substrate, poly (ADP-ribose) polymerase-1 (PARP-1), to the characteristic apoptotic fragment. These data indicate that through the coordinated regulation of membrane, mitochondrial and signaling events, B18L executes cancer cell death and thus has the potential to be developed into a potent and selective anti-cancer compound.

## 1. Introduction

For a long time, chemotherapy was the standard treatment for metastatic and drug resistant cancers. However, cancer therapy has advanced from use of chemotherapeutic agents to therapies with improved cancer cell selectivity, such as those that target different parts of the host immune system [1]. As a result, the identification of novel cancer driver genes and targets that are expressed in specific tumor types is critical in anti-cancer research. Thus, considerable resources have focused on agents that target growth factor receptors and now some host immune factors that regulate signaling and inflammation but not those that target cancer cell membranes.

Our research group has been involved in the development of BST-2-based inhibitors of adhesion-driven processes in cancer cells [2]. BST-2 is an innate immunity, interferon inducible, multifunctional protein that has important roles in inhibiting virus release [3,4,5,6], promoting cell to cell viral spread [7] and intracellular signaling [8]. BST-2 also plays key role in promoting oncogenic processes in many cancers, including breast cancer and its expression in these cancers is elevated relative to known cancer markers [9]. Indeed, the levels of BST-2 is high in many breast cancer cell lines [2] and its expression is regulated in part by DNA methylation, where BST-2 expression patterns in tumors and cancer cells correlate with hypomethylated BST-2 DNA [10]. Our published characterization and functions of BST-2 highlighted a huge potential for inhibiting BST-2 in cancer cells as an attractive anti-cancer strategy. Therefore, this manuscript presents additional findings documenting targeting BST-2 as a promising strategy to treat breast cancer.

We developed the first (B49 and B49Mod1) [2] and second (B18) [11] generations of BST-2-based peptide series with anti-adhesion activities. Although B18 inhibits cancer cell adhesion, it does not effectively kill cancer cells within a 24-h experimental window. B18 bears important structural features such as its hydrophobicity and hydrophobic moment as well as presence of negatively charged and membrane interacting residues that may be substituted to increase peptides net positive charge. These features make B18 amenable to modification and were manipulated to design 18-mer peptidomimetics in which structural modifications were performed to optimize anti-cancer activity.

Five 18-mer peptidomimetics (B18K, B18KL, B18KA, B18L and B18I), each containing a variation of features modified from B18 were evaluated for their cytotoxic activity. The peptidomimetics were tested against a series of breast cancer cell lines representing different cancer subtypes, including drug-resistant cell lines. Based on the cytotoxicity data, B18L, a cationic α-helical peptidomimetic was chosen as a lead candidate for structural and mechanistic characterization.

## 2. Results

### 2.1. Design of B18-Based Peptidomimetics

B18 peptide was the result of structure activity relationship (SAR) studies on B49Mod1 [2]. B18 has a net negative charge of –2 at physiological pH, inhibits cell to cell and cell to extracellular matrix (ECM) adhesion, with subtle polyfunctional cytotoxic effects on cancer cells [11]. To develop peptides with significant cytotoxicity toward cancer cells, we modified B18 through sequence substitution and created five B18-derived peptidomimetics named B18K, B18KL, B18KA, B18L and B18I (Table 1).

### 2.2. Design of B18K—Role of Polar Positive Amino Acids Alone

Table 1 displays the sequences of the B18-derived peptidomimetics and the sequential changes. It has been reported that anticancer agents with positive charge will target cancer cells effectively [12]. Therefore, we replaced the Asp (D) and Glu (E) polar negative residues of B18 with Lys (K) residues to generate a polar positive peptidomimetic that we named B18K. While B18K bears a net positive charge of +2 in comparison to B18 with a net negative charge of −2, the two compounds have similar hydrophobicity (H) and hydrophobic moment (µH) as shown in Table 1. Furthermore, the changes in B18K did not lead to increased cytotoxic activity on cancer cells except for the HER2+ cell line, SKBR3 (Table 2).

### 2.3. Design of B18KL and B18KA—Role of Hydrophobic Amino Acids

Since increasing the compound charge from net negative to net positive did not increase cytotoxicity, we generated B18KL by replacing B18K A10 and N13 with L10 and L13. These changes increased the compound’s H and µH from 0.326 and 0.224 to 0.531 and 0.315, while maintaining a net positive charge of +2 (Table 1). Although these alterations increased B18KL’s H and µH, the changes did not improve compound cytotoxicity (Table 2). However, substitutions of B18KL D4, E6, Q8 and T15 with K4, K6, K8 and K15 increased compound’s net charge to +3, while replacing A10, N13 and M17 with L10, L13 and L17 or V5, T11, H14 and V16 with A5, A11, A14 and A16 increased compound’s µH. Alanine (Ala, sidechain R = methyl) was used for substitution because of its non-bulky, chemically inert, methyl functional group that is generally equivalent to simply truncating a side chain back to the beta carbon, which is the first side chain atom [13]. These changes resulted in compound B18KA with a net positive charge of +4 (higher than B18KL), H of 0.357 (lower than B18KL) and µH 0.375 (higher than B18KL). Analysis of the cytotoxic effect of B18KA showed a significant improvement over B18KL. The inhibitory concentration of B18KA that killed 50% (IC_50_) of basal MDA-MB-468, claudin-low MDA-MB-231, luminal A/B ZR-75-1, luminal A T47D and HER2 SKBR3 cells were 135.6 ± 9.3 µM, 83.7 ± 20.1 µM, 58.9 ± 2.0 µM, 78.5 ± 20.9 µM and 43.5 ± 11.5 µM respectively (Table 2). Although the IC_50_ was high, these substitutions indicated that structural alterations that maintains a balance between positive charge and increased H/µH may result in improved cytotoxicity against some cancer cells.

### 2.4. Design of B18L as a Compound Lead—Role of Branched-Chain Amino Acids (BCAAs)

The substitutions in B18KA revealed that balancing compound charge, H, and µH may result in engineering an effective agent that is cytotoxic against cancer cells. Thus, we sought to assess how substitutions with any of the three BCAAs— valine, leucine, and isoleucine, which are among the most hydrophobic amino acids would affect B18KA’s cytotoxic property. BCAAs play crucial roles in determining protein structures since they regulate the interaction of the transmembrane domains of membranous proteins with phospholipid bilayers. Although the three BCAA share some similarity, they play different roles in proteins, their side-chains differ in size, shape, H and their structures are different. Thus, their behaviors are also different. Valine and isoleucine have preference for β-structures and leucine prefers α-helix. Since B18KA with higher hydrophobicity and positive net charge of +4 showed some cytotoxicity compared to B18KL, we embarked on evaluating the effect different BCAA may have. Thus, we made B18KA substitutions with isoleucine (prefers β-structures) and leucine (prefers α-helixes) among others. Substitutions of Phenylalanine for Leucine (F2L), Glutamine for Glycine (Q3G) and lysine for Leucine (K6L) in B18KA resulted in B18L bearing a net positive charge of +3, H of 0.513 and µH of 0.523. Leucine for Isoleucine (L2I and L6I) substitutions resulted in B18I with a net positive charge of +3, H of 0.541 and µH of 0.547. These data indicate that Leucine and Isoleucine substitutions may have significant effects on the functions of the compound.

Therefore, we evaluated the effect of these amino acid substitutions on cancer cell viability. Compared to B18KA that did not have effect on MDA-MB-468 or had subtle effects on the viability of MDA-MB-231, T47D, ZR-75-1 and SKBR3 cells, we observed significant improvement on the cytotoxic effect of B18L and B18I on these cells (Table 2). Of interest and in contrast to B18KA is the ability of B18L and B18I to kill MDA-MB-468 cells of the basal subtype of breast cancer at IC_50_ of 12.4 ± 1.4 µM and 16.6 ± 1.5 µM respectively. Similarly, while B18KA killed SKBR3 HER2 cells at high IC_50_ of 43.5 ± 11.5, the IC_50_ at which B18L and B18I killed the cells were 7.2 ± 1.0 and 18.1 ± 4.7 µM, which is much lower than that of B18KA (Table 2). Although both B18L and B18I have comparable cytotoxic effects on the cells tested, B18L is the only compound that killed T47D cells at IC_50_ of 17.5 ± 6.5 µM compared to the effect of B18I that was unable to kill T47D (IC_50_ of >148.5 µM).

### 2.5. B18L As a Compound Lead—Activity Toward Drug-Resistant Breast Cancer (DRBC) Cells

Given the polyfunctional activities of B18 analogs against varied subtypes of breast cancer cells (Table 2), we assessed the effects of the compounds on DRBC cells since drug resistance is one of the main causes of failure in cancer treatment. We focused on Tamoxifen resistance because ~80% of all breast cancers are estrogen receptor positive (ER+) cancers. ER+ breast cancer subtype is mostly treated with Tamoxifen and about 50% of ER+ tumors do not respond to therapy, resulting in DRBC. Only B18L displayed cytotoxic activity against drug-resistant (MCF7-G11-TR1, MCF7-G11-TR5) as well as drug-sensitive (MCF7) breast cancer cell lines at low single digit IC_50_ of 3.8 ± 0.3 µM to 6.2 ± 0.8 µM (Table 3). Remarkably, IC_50_ of 3.8 ± 0.3 that kills MCF7-G11-TR5 cells is much lower than the concentration (IC_50_ of 5.9 µM) that kills MCF7 cells. It is noteworthy that the IC_50_ of 10.8 ± 2.0 µM to 16.1 ± 4.9 µM of B18I required to kill both drug-resistant and drug-sensitive cell lines respectively is much lower than 56.4 ± 5.0 µM to 61.8 ± 5.1 µM of B18KA (Table 3). This screening of the compounds against drug-resistant and drug-sensitive breast cancer (DSBC) cell lines clearly demonstrates that B18L is overall the most active compound.

### 2.6. Structure of B18L

**Figure 1:**
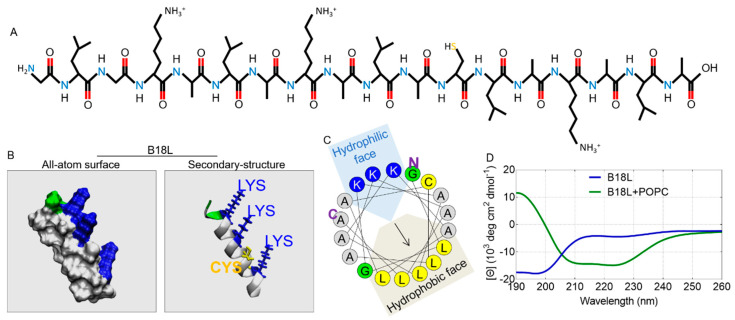
Structural properties of B18L: (**A**) Chemical structure of B18L. (**B**) PEP-FOLD3 prediction for B18L. Left panel shows all-atom surface and right panel shows secondary structure with four residue side chains (LYS and CYS). Color code: blue: basic residue, green: polar residue, white: non-polar residue and yellow: CYS residue. (**C**) Helical wheel projection of B18L re-drawn based on Heliquest, **N**: N-terminus and **C**: C-terminus. (**D**) Circular dichroism (CD) spectrum of B18L in water or 1-palmitoyl-2-oleoyl-glycero-3-phosphocholine (POPC) lipid solution.

The chemical structure of B18L as predicted by PepDraw (Wimley Laboratory, Tulane University, New Orleans, LA, USA) is a linear molecule without branches (Figure 1A). Prediction with PEP-FOLD3 [14] show the all-atom surface structure as rod-like (Figure 1B, left) while the secondary structure is helix-like with three charged residues (lysine, LYS, K) facing one side (Figure 1B, right). The structural properties of B18L was also assessed by helical wheel projection, which shows three lysine residues on the hydrophilic face and five leucine (L) residues on the opposite hydrophobic face (Figure 1C). These structural features indicate that B18L is an amphiphilic compound. Furthermore, circular dichroism (CD) measurements of B18L (Figure 1D) show a folding event in peptide structure from random coil to helix when transferred from aqueous buffer (blue line) to the lipophilic solvent (green line) with 8% to 42% increase in helicity. In addition, β-strand and irregular structural features decreased from 22% and 75% to 21% and 47% respectively. Although not the focus of this manuscript, it is reasonable to assume B18L will form helical structure in different peptide/lipid ratios [15,16]. While supporting the projected B18L structure, the CD spectra shows that structure is an important feature of B18L, and the environment of the compound is important to compound structure.

### 2.7. B18L Binds BST-2 Protein

**Figure 2:**
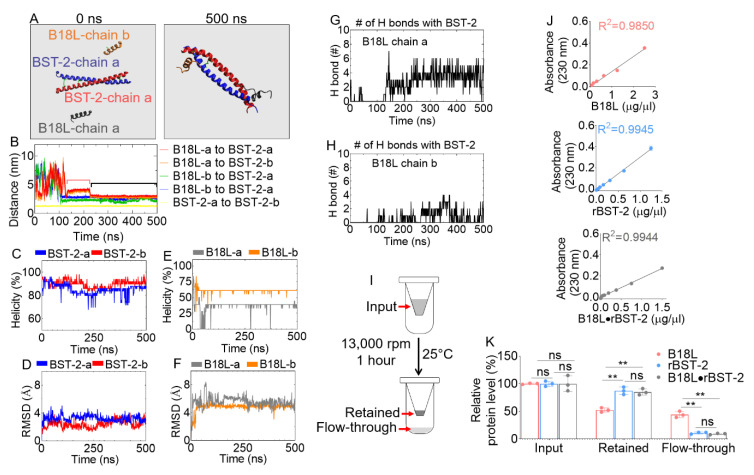
Interaction of B18L with BST-2: (**A**) Initial (t = 0, left) and final (t = 500 ns, right) snapshots of two B18L chains with BST-2 protein. (**B**) Evolution of center of mass (COM) distance between each chain in the MD system. Pink and black brackets represent two different binding positions. (**C**) Helicity and (**D**) RMSD of BST-2 as a function of time. (**E**) Helicity and (**F**) RMSD of B18L as a function of time. (**G**) Formation of hydrogen bonds as a function of time for B18L chain a with BST-2. (**H**) Formation of hydrogen bonds as a function of time between B18L chain b and BST-2. (**I**) Schematic of in vitro B18L•BST-2 interaction in vitro assay. (**J**) Standard curve used for protein concentration extrapolation from UV absorbance analysis. Linear regression and R-square values were obtained from the GraphPad Prism analysis. (**K**) Compound concentrations following extrapolation from UV absorbance. Unpaired t-test with Welch’s correction was used to determine the differences between the treatments. ** *p* < 0.01 and ns = not significant. B18L-a is the same as B18L chain a, B18L-b is the same as B18L chain b; same for BST-2-a and BST-2-b. Experiment was repeated at least three times, with similar results.

Since B18L is a derivative of BST-2-based peptides B49B49Mod1 [2] and B18 [11], we assessed its interaction with the extracellular domain of BST-2 using two different assays—molecular dynamics (MD) simulation and in vitro binding of B18L with recombinant BST-2 (rBST-2) [9] based on ultraviolet (UV) absorbance at 230 nm. MD simulation of two chains of B18L (chains a and b) and BST-2 (chains a and b) in 0.15 M KCl neutral aqueous solution were performed for 500 ns. BST-2 chains a and b were linked with disulfide bonds. The peptides were initially placed in the same distance (5 nm) to the center of mass (COM) of BST-2 protein (Figure 2A, left). The 500 ns snapshot illustrates the binding of B18L chains a and b on to BST-2 protein (Figure 2A, right). Since the distance between molecules is the most straight forward way to determine whether interaction is occurring [17], the evolution of distances between the COM of each B18L chains to the COM of each BST-2 chain was determined (Figure 2B). From this analysis, we observed that B18L chains a and b (Figure 2B, red and orange lines) bind BST-2 within the first 150 ns simulation (Figure 2B). The binding occurred for B18L chain a from 130 ns to 500 ns and from 105 ns to 500 ns for B18L chain b (Figure 2B). B18L chain a switched position from 130–230 ns (average distance ~4.1 nm, pink bracket) to 230–500 ns (average distance ~3.1 nm, black bracket), implying two potential binding sites for B18L onto BST-2. The COM distance between the BST-2 chains is stable as expected (Figure 2B, yellow line), perhaps due to disulfide bonds linkage. Additional structural information for BST-2 and B18L interactions were derived from helicity and root-mean-square deviation (RMSD) calculations as a function of time. The calculations show that both chains of BST-2 maintain helicity above 70% (Figure 2C) but the RMSD of BST-2 fluctuated within 4 Å (Figure 2D). Unlike BST-2, B18L helicity was ~38% and ~61% (Figure 2E) for chains a and b, while the RMSD of B18L chain a showed more fluctuation compared to chain b (Figure 2F). These data imply that the structure of B18L is stable within the simulation time. The presence of hydrogen bonds between B18L chain a and BST-2 (Figure 2G) or between B18L chain b and BST-2 (Figure 2H) as a function of time provides confirmation of the existence of binding interaction. Altogether, the MD simulation results suggest that B18L binds BST-2 within a short time and that the binding interaction between the two molecules is stable.

To validate the MD simulation data, we used UV absorbance based assay [18] to assess the binding affinity of B18L to BST-2 (B18L•BST-2). Following incubation of B18L with rBST-2 or controls (B18L alone and rBST-2 alone) at 37 °C for 45 min, each compound or the B18L•BST-2 mixture was placed in centrifugal filters and spun at 13,000 rpm for 1 h (Figure 2I). Absorbance of compounds (i) prior to addition to the filter (input), (ii) on the apical side of the filter (retained), (iii) in the collection tube (flow through) were obtained and converted to compound concentration using the standard curves (Figure 2J). The results (Figure 2K) show that 52%, 78%, 70% of B18L, BST-2 and B18L•BST-2 respectively were retained on the apical side of the filter, while 45%, 21%, 10% of B18L, BST-2 and B18L•BST-2 respectively were in the flow-through. These data indicate that most B18L complexed with rBST-2 to form a B18L•BST-2 compound that resulted in the least (10%) flow-through (Figure 2K). 

### 2.8. B18L Is Less Toxic to Normal Human Cells

**Figure 3:**
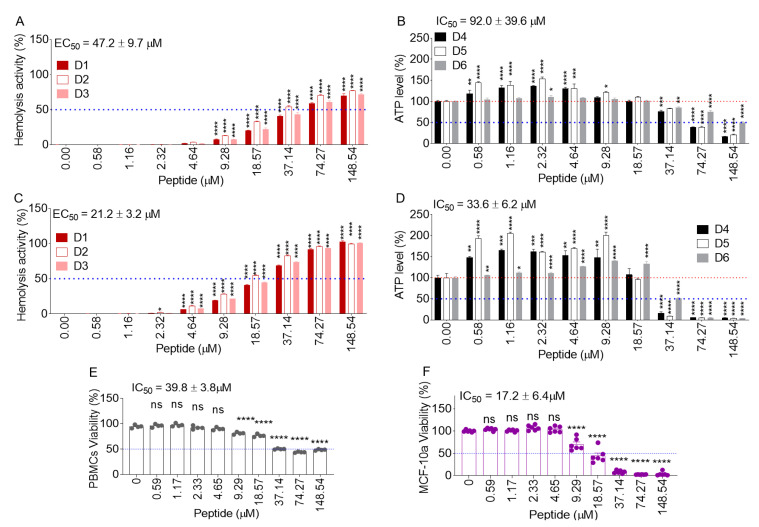
Response of erythrocytes and peripheral blood mononuclear cells (PBMCs) to B18L: Hemolytic and cytotoxic effects of B18L respectively on erythrocytes and PBMCs at 0.75 h (**A** and **B**) and 24 h (**C** and **D**) post treatment. Cytotoxic effects of B18L on PBMCs was assessed using the ATP assay. (**E**) MTT viability assay of PBMCs after treatment with B18L for 24 h. (**F**) MTT viability assay of MCF-10a cells after treatment with B18L for 24 h. Each erythrocyte and PBMC assay used three independent healthy donors (**D**) D1 to D3 were used for hemolysis while D4 to D6 were used for PBMC viability. Error bars represent the standard error of the mean (SEM) of at least four replicates. Ordinary one-way ANOVA test (Dunnett’s correction) was used to determine the differences between the treated groups as compared to vehicle treatment. * *p* < 0.05, ** *p* < 0.01, *** *p* < 0.005, **** *p* < 0.001; not significant was not shown for clarity for panels A-D and shown as ns for panels E-F. Experiment was repeated at least three times, with similar results.

To determine whether B18L is selectively toxic to cancer cells, the effect of B18L was evaluated in healthy human erythrocytes and peripheral blood mononuclear cells (PBMCs) isolated from the blood of healthy donors. PBMCs are usually used to evaluate the cytotoxicity of natural or synthetic products to normal cells [19]. Thus, the hemolytic effect of B18L on erythrocytes and the cytotoxic effect of B18L on PBMCs were evaluated early (45 min) post treatment. The effective concentration of B18L that resulted in 50% hemolysis (EC_50_) when compared with that of the positive control was 47.2 ± 9.7 μM, while the IC_50_ on PBMCs was = 92.0 ± 39.6 μM. These data suggest that adverse hemolytic (Figure 3A) and cytotoxic (Figure 3B) effects were not detected against erythrocytes and PBMCs respectively at concentrations that killed cancer cells (Table 2 and Table 3).

To confirm that reduced toxicity to erythrocytes and PBMCs was not a consequence of delayed kinetics, the hemolytic effect of B18L on erythrocytes and the cytotoxic effect of B18L on PBMCs were evaluated at 24 h post treatment. EC_50_ and IC_50_ of 21.2 ± 3.2 µM and 33.6 ± 6.2 μM were observed for erythrocytes (Figure 3C) and PBMCs (Figure 3D) respectively at the 24 h time point. Noteworthy is the donor (D) variability in erythrocyte and PBMCs responses to B18L. The variability was most notable in the PBMC ATP assay, where increased ATP levels were detected in D4 and D5 at low peptide concentrations. This difference is clearly a donor dependent effect that requires additional investigation in a different study. We confirmed the high IC_50_ (39.8 ± 3.8) of B18L on PBMC (D6) using the MTT assay (Figure 3E). However, in the MTT assay, we did not observe the increase in cell viability as shown in the ATP assay in Figure 2C,D. We also tested the response of a normal breast cell line, MCF-10a, to B18L and found that the IC_50_ of 17.2 ± 6.4 µM for MCF-10a was higher than that of MCF7-G11-TR5 (3.8 ± 0.3 µM) (Figure 3F). Together, these results suggest that B18L is less toxic to normal human cells than the drug resistant cancer cell line―MCF7-G11-TR5.

### 2.9. B18L Causes Early DRBC Apoptotic Cell Death and Triggers Intracellular Signal Transduction

**Figure 4:**
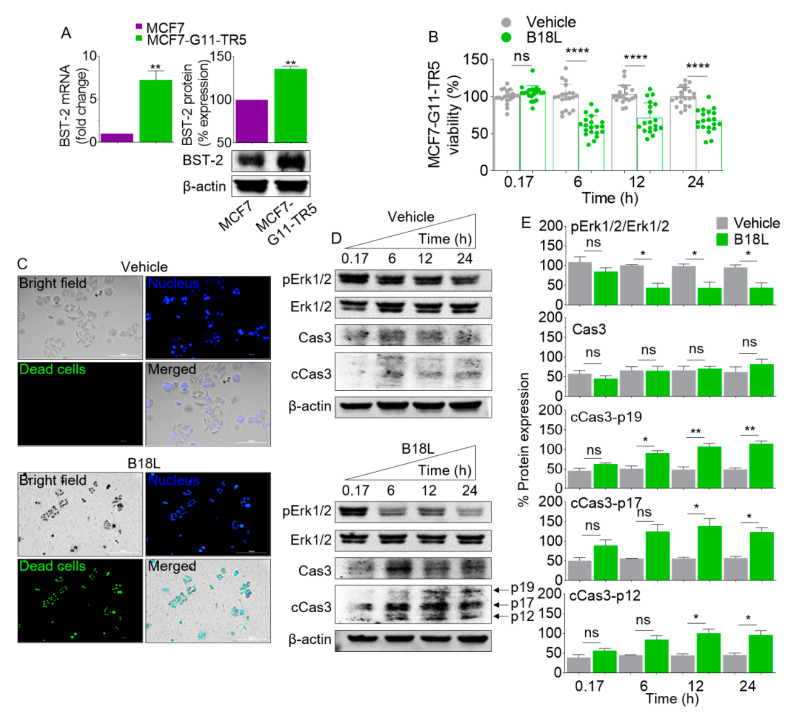
Time-dependent response of MCF7-G11-TR5 cells to B18L: (**A**) Levels of BST-2 mRNA and protein in MCF7 and MCF7-G11-TR5 cells. Difference in BST-2 is presented as fold change relative to MCF BST-2 set to 1 for mRNA and 100 for protein. (**B**) Viability of MCF-G11-TR5 B18L cells following treatment with B18L at different (0.17, 6, 12 and 24 h) time points. Error bars represent the SEM of at least 20 replicates. Unpaired t-test with Welch’s correction was used to determine the differences between vehicle and B18L treatment at each time point. **** *p* < 0.001 and ns = not significant. (**C**) Analysis of B18L-mediated killing of MCF7-G11-TR5 cells using the EasyProbes cell viability assay at 24 h after treatment. Representative 10× images acquired using the Lionheart FX automated microscope. Blue: live cell stain. Green: dead cell stain. Scale bar: 200 μm. (**D**) Protein expression levels of pErk1/2, Erk1/2, Caspase 3 (Cas3) and cleaved Caspase 3 (cCas3) under vehicle/B18L treatment and (**E**) Quantitation of protein expression levels for three independent replicate blots for the proteins shown in panel C. Unpaired t-test with Welch’s correction was used to determine the differences between vehicle and B18L treatment at each time point. * *p* < 0.05, ** *p* < 0.01 and ns = not significant. All the replicate blots and original membranes are shown in Figures S1–S4. Densitometry readings/intensity ratio is shown in Tables S1,S2. Experiment was repeated at least three times, with similar results.

Data in Table 3 show the cytotoxic effect of B18 analogs on DRBC MCF7-G11-TR5 cells. Based on this data, B18L is the most potent analog that kills MCF7-G11-TR5 cells. Since B18L killed MCF7 and MCF7-G11-TR5 cells at low IC_50_ of 5.9 ± 0.5 and 3.8 ± 0.3 (Table 3) and B18L binds BST-2, we assessed the levels of BST-2 RNA and Protein. As expected, MCF7-G11-TR5 cells has relatively higher levels of BST-2 RNA and protein compared to parental MCF7 cells (Figure 4A). To begin to understand the kinetics of B18L-mediated killing of MCF7-G11-TR5 cells, we conducted a time-dependent assessment of cytotoxicity on the cells. The data show that 3.8 μM of B18L caused significant cell death as early as 6 h post treatment with no significant change in cell viability at 12 and 24 h post compound addition (Figure 4B). Microscopically, B18L-treated cells exhibit impaired membrane resulting in fluorescently (green) labeled cells (Figure 4C). Since vehicle treated cells did not fluoresce, this result indicates that B18L impaired cell membrane integrity. The loss of cell viability is accompanied by a decrease in the levels of phosphorylated extracellular signal-regulated protein kinases 1 and 2 (Erk1/2) as early as 6 h post treatment (Figure 4D, bottom, Figure 4E). Furthermore, we observed constitutive presence of the 32 kDa procaspase-3 (Cas3) protein in B18L-treated cells (Figure 4D, bottom, Figure 4E). Cas3 protein is cleaved into 19, 17 or 12 kDa subunits [20,21,22,23,24]. We observed differences in the intensities of the cleavage products and we quantified the different subunit products. While there was slight labeling of the 19, 17 and 12 kDa subunits of cCas3 in vehicle treated cells, all subunits have increased labeling intensities in B18L-treated cells (Figure 4D, bottom, Figure 4E). Collectively, these data indicate that B18L-mediated cell death mechanism is an early event.

### 2.10. Structural Dynamics of B18L on the Cell Membrane as Assessed By Molecular Dynamics (MD) Simulations

**Figure 5:**
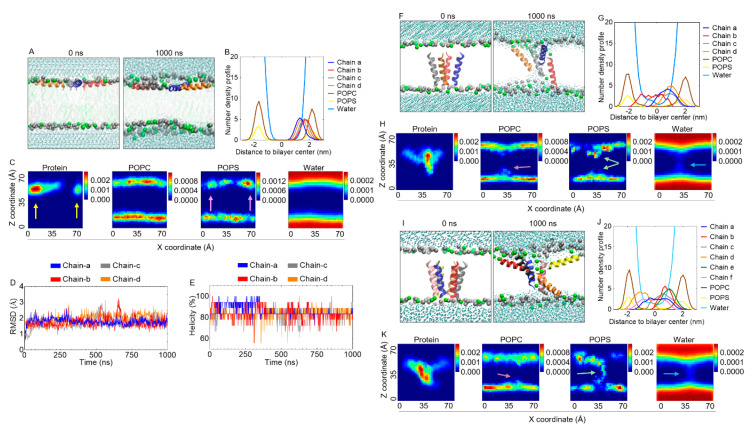
Molecular dynamics (MD) simulation of the interaction of B18L with model POPC/POPS membrane: (**A**) Initial (left, t = 0) and final (right, t = 1000 ns) snapshots of the interaction of B18L with POPC/POPS cell membrane. Color code: blue = B18L chain a, red = B18L chain b, gray = B18L chain c and orange = B18L chain d, silver particles = phosphate atom of POPC lipid, green particles = phosphate atom of POPS lipid, cyan dots = the oxygen atom of water. (**B**) One-dimensional (1D) and (**C**) two-dimensional (2D) density of the simulation system. Yellow arrows: enrichment of B18L molecules. Pink arrows: enrichment of POPS lipid heads. (**D**) RMSD and (**E**) helicity of each individual B18L peptide as a function of time for surface status. (**F**) Initial (left, t = 0) and final (right, t = 1000 ns) snapshots of four B18L peptides interaction with POPC/POPS cell membrane. Color code: same as Figure 5A. Lipid tails were not shown for clarity. (**G**,**H**) 1D and 2D density of the simulation system. Pink arrow: bent POPC lipid heads. Light green arrows: bent POPS lipid heads. Light blue arrow: water channel. (**I**) Initial (left, t = 0) and final (right, t = 1000 ns) snapshots of six B18L peptides interaction with POPC/POPS cell membrane. Color code: yellow: B18L chain e, pink: B18L chain f and the rest are the same as Figure 5A. Lipid tails were not shown for clarity. (**J**,**K**) one dimensional (1D) and two-dimensional (2D) density of the simulation system. Color codes for the arrows are as described for Figure 5H.

The data in Figure 4B showing that B18L-mediated cytotoxic effect on MCF7-G11-TR5 cells occurred as early as 6 h suggest potential B18L interaction with the cell membrane. Hence, we used MD simulations to gain insight into the interaction of B18L with the cell membrane at the atomic level. To mimic the mammalian cancer cell membrane, we used a 3:1 ratio of 1-palmitoyl-2-oleoyl-glycero-3-phosphocholine/1-palmitoyl-2-oleoyl-sn-glycero-3-phospho-L-serine (POPC/POPS) lipid bilayer mixture. For initial simulation, four B18L molecules were surface adsorbed on the upper leaflet of the lipid bilayer. Snapshots of the systems at time 0 and 1000 ns show that the B18L molecules remained on the surface of the membrane for the duration of the simulations (Figure 5A). The density profile analysis of the last 300 ns trajectory suggests that B18L molecules were mostly distributed ~0–2 nm to lipid bilayer center (Figure 5B). Two-dimensional (2D) analysis shown in Figure 5C further confirm that the B18L molecules remained on the membrane surface. Specifically, the far left 2D plot show the distribution of peptides as pointed by yellow arrows and the middle left 2D plot shows the distribution of POPC lipid heads. Since the position of peptides is lower than POPC lipid heads, this indicates the peptides are binding the membrane by interacting with both lipid heads and lipid tails. Interestingly, we observed enrichment of POPS lipid heads at the upper leaflet where B18L chains aggregated (Figure 5C, pink arrows on the middle right 2D plot), an indication that B18L may induce rearrangement of PS lipids by electrostatic attraction. Water molecules were separated by the lipid bilayer as shown in the far right 2D plot (Figure 5C), indicating there is no water diffusion or membrane rupture event during the simulation. Number of hydrogen bonds between B18L and different component of the systems (H_2_O/POPC/POPS, Figure S5A) show that all the B18L chains were able to form hydrogen bonds with H_2_O, POPC or POPS. Chain a has the least number of hydrogen bonds with H_2_O (Figure S5A, H_2_O row far right). This can be explained by deep localization of the peptide in the bilayer (Figure 5B). Of note, chain a and chain b formed relatively less hydrogen bonds with POPC but more hydrogen bonds with POPS compared to chain c and chain d (Figure S5A, POPC row far right and POPS row far right). This difference in the number of hydrogen bonds may imply additional interaction between the peptides (Figure S5B, chain a—chain d and chain b—chain c). Further analysis suggests that B18L stably binds the membrane over time as indicated by the RMSD (Figure 5D) and helicity (Figure 5E) data.

Given that transmembrane insertion of the parental B18 revealed the potential for water channel formation [11], we assessed the effect of B18L on the membrane when transmembrane-inserted. Four monomers chains of B18L were transmembrane inserted into POPC/POPS mixed lipid bilayer and simulated for 1000 ns. Initially, all B18L chains were perpendicular to the membrane surface (Figure 5F, left), after 1000 ns, B18L chains a and d adopted parallel orientation while chains b and c remained in the transmembrane (Figure 5F, right). This is not surprising because peptides can interact with cell membrane when parallel to membrane surface or perpendicular to membrane surface (transmembrane) [25,26,27]. The last 300 ns one-dimensional (1D) and two-dimensional (2D) density profiles (Figure 5G,H far left) showed consistent result as Figure 5F. In addition, lipid heads and water channel were present in the center of lipid bilayer (Figure 5G,H middle left-far right), indicating potential rupture of lipid bilayer and pore formation. Noteworthy is that the structure of B18L chains were even more stable than the surface state as indicated by the RMSD (Figure S6A) and helicity (Figure S6B) evolutions. The number of hydrogen bonds between B18L and POPS lipid in the transmembrane state (Figure S7A) is higher than in the surface state, indicating that electrostatic interaction may play a role in stabilizing the pore structure. The formation of hydrogen bonds between neighboring B18L chains (Figure S7B) indicate that intermolecular interaction between B18L chains may play a role in pore structure stability.

Since two of four B18 chains (chains a and d) tend to move to the surface (Figure 5F,G), we sought to analyze B18L’s pore structure with increasing peptide chains. Thus, six monomer chains of B18L were initially placed inside the POPC/POPS lipid bilayer to check the pore structure. The simulation showed that when six B18L monomer chains were initially placed perpendicular to the membrane (Figure 5I, left), four of six chains changed their orientation and tilted into the surface of the membrane (Figure 5I right, Figure 5J,K far left). The pore structure is accompanied by a much larger water channel compared to that formed by four monomer chains (Figure 5J,K far right). POPC lipid heads bent a little into the bilayer center (Figure 5K, middle left) which is similar to the four monomer chains but POPS lipid heads (Figure 5K, middle right) persistently bent into the lipid bilayer, implying that more POPS lipids may be attracted by B18L. The RMSD evolution (Figure S8A) showed that chains b–f are relatively stable within 1000 ns simulation (~0.2 nm) while chain a adjusted its structure at the first 250 ns with a higher RMSD (~0.3 nm) and remained stable for the rest of the simulation (~0.2 nm). The helicity evolution (Figure S8B) also agreed with the structure change, as chain a helicity changed from ~70% at 0–250 ns to ~90% at 250–1000 ns and chains b–f kept the helicity around 80–90%. Formation of hydrogen bonds are similar to the previous observation, for example, four of six peptides formed more hydrogen bonds with POPS lipid (Figure S9A), indicating the dominant effect of electrostatic attraction. Hydrogen bonds between neighboring B18L chains also showed intramolecular interactions during the simulation (Figure S9B). Together, these MD simulations suggest a possible membranolytic mechanism of action for B18L.

### 2.11. B18L Impairs Cancer Cell Membrane Integrity

**Figure 6:**
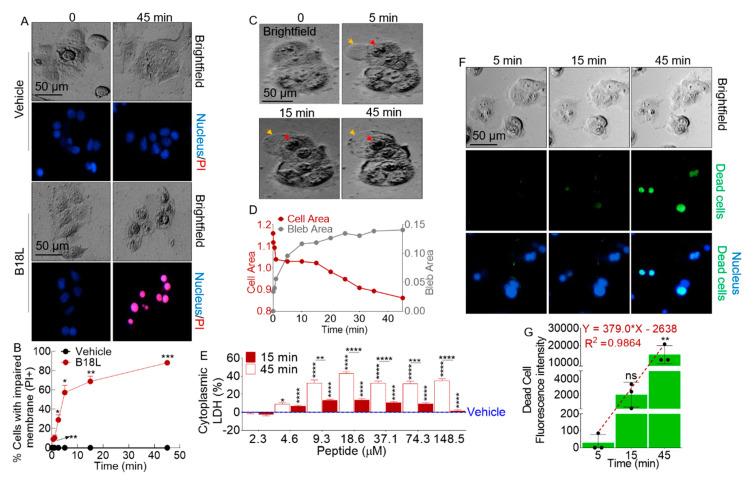
Membranolytic effects of B18L: (**A**) DAPI/PI staining of MCF7-G11-TR5 treated with vehicle or B18L at 0 and 45 min. Fluorescence images were manually obtained at 10× using the Lionheart FX automated microscope. Scale bar: 50 μm. (**B**) Quantification of cells with impaired membrane at 0.5, 1, 2.5, 5, 15, 45 min. Five 10× fields of view were analyzed per time point. Two-tailed Welch’s t test was used to compare the difference between vehicle and B18L at each concentration. *** *p* < 0.005, ** *p* < 0.01, * *p* < 0.05 and nonsignificant was not shown for clarity. (**C**) Representative live cell images of membrane blebs captured at 0, 5, 15, and 45 min. 10× kinetic images were acquired using the Lionheart FX automated microscope. Orange arrows indicate membrane blebs and red arrows indicate the nucleus of blebbing cells. Scale bar: 50 μm. (**D**) Quantification of area of the cell versus area of the bleb as a function of time. (**E**) LDH leakage assessed in the supernatant of MCF7-G11-TR5 cells treated with various concentrations of vehicle or B18L after 15 min or 45 min treatment. Two-tailed Welch’s t test was used to compare the difference between vehicle and B18L at each concentration. Ordinary one-way ANOVA test (Dunnett’s correction) was used to determine the differences between the treated groups as compared to vehicle treatment. **** *p* < 0.001, *** *p* < 0.005, ** *p* < 0.01, * *p* < 0.05 and nonsignificant was not shown for clarity. (**F**) Representative live/dead cell stain images of vehicle and B18L treated cells revealing free amines (green florescence) and nucleus (blue fluorescence). 10× kinetic images were taken using the Lionheart FX automated microscope. Scale bar: 50 μm. (**G**) Quantification of dead cell fluorescence intensity at different time points. The fitted linear equation and R-square value were obtained by GraphPad Prism analysis. Error bars represent SEM of at least three replicates. Ordinary one-way ANOVA test (Dunnett’s correction) was used to determine the differences between the treated groups as compared to vehicle treatment. ** *p* < 0.01 and ns = not significant. Experiment was repeated at least three times, with similar results.

Given the early (6 h) cytotoxic effect of B18L (Figure 4B) and its predicted membrane interaction (Figure 5), we evaluated the effect of B18L on necrotic cell death of MCF7-G11-TR5 cells using propidium iodide (PI) uptake assay. Compared to vehicle, MCF7-G11-TR5 cells internalized PI (red fluorescence) as early as 0.5 min after exposure to B18L (Figure S10A). Uptake of PI increased over time up to 45 min (experiment end time) post treatment in B18L treated cells (Figure 6A and Figure S10A). Quantification of PI positive cells show that 10.2, 29.0, 57.2, 68.8 and 88.2% of cells internalized PI at 0.5, 1, 2.5, 5, 15, and 45 min post addition of B18L (Figure 6B). These data suggest that B18L induces plasma membrane alteration and that cells display loss of plasma membrane integrity. To monitor the effects of B18L at the morphological level, we performed time-lapse imaging of MCF7-G11-TR5 cells exposed to B18L or vehicle. Incubation with B18L but not vehicle, induces massive cell blebbing, together with progressive formation of electron dense material around the cell membrane (Figure 6C and Figure S10B), suggestive of necrotic events. We observed time-dependent increases in the area of the blebs (Figure 6C and Figure S10B) that is accompanied by decrease in the area of the cells (Figure 6D) as membrane blebs increase. Necrotic cells release endogenous molecules, such as lactate dehydrogenase (LDH). As shown in Figure 6E, exposure to B18L results in a time-dependent increase in LDH release into the culture media, confirming membrane damage. Release of LDH was concomitant with cell death as shown by the increase in the level of free amines (green florescence) following treatment (Figure 6F,G). Collectively, these data indicate that B18L may induce rapid necrosis of MCF7-G11-TR5 cells via membranolysis.

### 2.12. B18L Potentiates Phosphatidylserine (PS) Externalization in the Plasma Membranes

**Figure 7:**
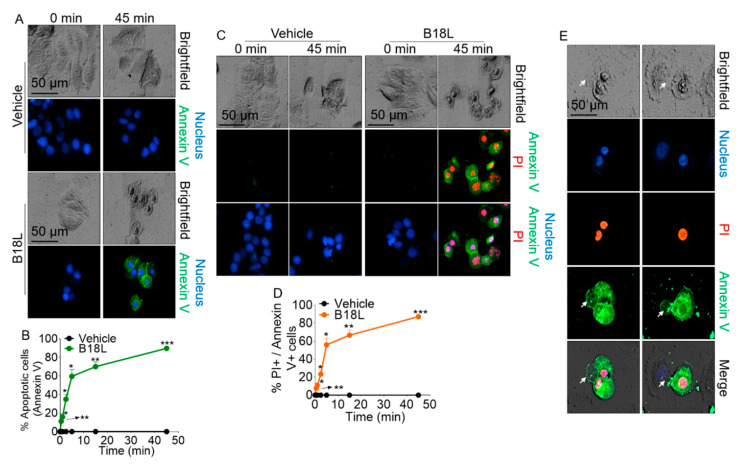
Effect of B18L on Annexin V binding to Phosphatidylserine (PS). (**A**) Pattern of 4′,6-diamidino-2-phenylindole (DAPI) and Annexin V staining of MCF7-G11-TR5 cells following vehicle (upper) or B18L (lower) treatment at 0 and 45 min. Fluorescence images were manually obtained at 10× using the Lionheart FX automated microscope. Scale bar: 50 μm. (**B**) Quantification of cells in panel A exhibiting externalized PS designated as Annexin V+ cells. Five 10× fields of view were analyzed at 0.5, 1, 2.5, 5, 15 and 45 min. Two-tailed Welch’s t test was used to compare the differences between vehicle and B18L at each concentration. *** *p* < 0.005, ** *p* < 0.01, * *p* < 0.05 and nonsignificant was not shown for clarity. (**C**) Pattern of DAPI, PI and Annexin V staining of MCF7-G11-TR5 cells following vehicle (left) or B18L (right) treatment at 0 and 45 min. Fluorescence images were manually obtained at 10× using the Lionheart FX automated microscope. Scale bar: 50 μm. (**D**) Quantification of cells in panel C exhibiting internalized PI (PI+) and externalized PS (Annexin V+ cells). Five 10× fields of view were analyzed at 0.5, 1, 2.5, 5, 15 and 45 min. Two-tailed Welch’s t test was used to compare the differences between vehicle and B18L at each concentration. *** *p* < 0.005, ** *p* < 0.01, * *p* < 0.05 and nonsignificant was not shown for clarity. (**E**) Representative images of Annexin V+ membrane blebs. White arrows indicate Annexin V+ membrane blebs. Experiment was repeated at least three times, with similar results.

Phosphatidylserine (PS) is a major component of membrane bilayer and alteration in its distribution between the inner and outer leaflets is an important signal indicative of the physiological or pathophysiological state of a cell. Since we observed at the atomic level, enrichment of POPS lipid heads at the upper leaflet where B18L chains aggregated (Figure 5C, middle right), we became interested in determining the extent to which B18L is associated with changes in PS externalization at the cellular level. To quantify PS exposure, we used time-lapse microscopic analysis of fluorescently labeled Annexin V to evaluate the number of cells with externalized PS and to quantify the amounts of PS on the cell surface. Using Annexin V-GFP labeling, we found no significant increase in the number of Annexin V-positive MCF7-G11-TR5 cells after treatment with vehicle (Figure 7A, top two rows). However, treatment of MCF7-G11-TR5 cells with B18L (3.8 µM) resulted in a significant time-dependent increase in the number of cells with externalized PS (Figure 7A, bottom two rows). Increasing the treatment time of MCF7-G11-TR5 cells with B18L to 45 min, resulted in a significant increase in the number of cells with externalized PS (Figure 7A,B and Figure S11A). At 45 min of exposure of MCF7-G11-TR5 cells to B18L, 89.7% of cells were Annexin V-positive. At this later time point, vehicle did not induce externalization of PS in cells (Figure S11A and Figure 7B). In contrast to vehicle treated cells (Figure 7C, left two columns, Figure S11B), B18L treatment was also associated with significant increase in the number of PI-positive and Annexin V-positive cells during the exposure times (Figure 7C, right two columns, Figure S11B), resulting in 87.3% of cells being both PI and Annexin V positive (Figure 7D). Finally, we observed that B18L-induced membrane blebs contain PS as evidenced by Annexin V positive staining of membrane blebs (Figure 7E).

### 2.13. B18L Activates Integrin Alpha 5 and Beta 1 Expression and Induces Sustained Inhibition of Src and Erk1/2 Activation

**Figure 8:**
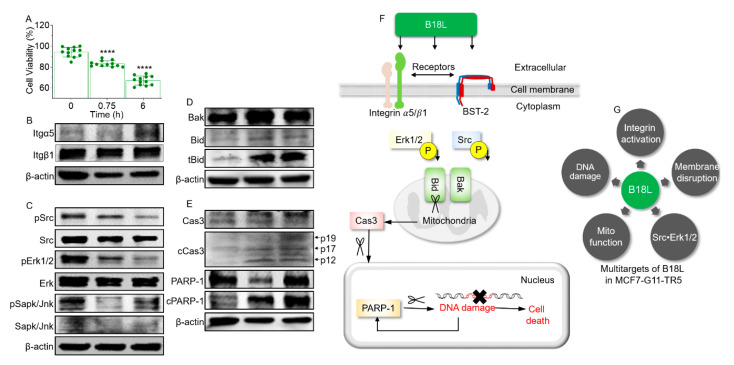
Intracellular signaling events in cells following treatment with B18L: (**A**) Cell viability following treatment with Vehicle (VEH) or B18L at 0.75 h or 6 h. Ordinary one-way ANOVA test (Dunnett’s correction) was used to determine the differences between the treated groups as compared to pre-treatment. **** *p* < 0.001. Western blot analysis of the effect of B18L on protein levels of (**B**) Integrins, (**C**) kinases, (**D**) BH3-only member of Bcl2 family of proteins, (**E**) Cas3 and PARP-1. β-actin was used as loading control. All replicate blots and original membranes are shown in Figures S12–S15. Densitometry readings/intensity ratio are in Table S3. (**F**) Cartoon of signaling molecules regulated by B18L. (**G**) Schematic of the multi-target effects of B18L on MCF7-G11-TR5. Experiment was repeated at least three times, with similar results.

Cellular stresses and stimuli induce cell death via activation of integrins (Itg) and various kinases. Association between the expression of Itg α5 and β1 have been made in cancer cells [28,29]. Thus, we examined the levels of Itg α5 and β1 following B18L treatment. As expected, 3.8 µM B18L decreased cell viability even as early as 0.75 h post (45 min) treatment (Figure 8A). The decrease in cell viability is accompanied by an early steady increase in Itg α5 but not Itg β1 levels (Figure 8B and Figure S12). Furthermore, we observed decreases in phosphorylated (p) Src (pSrc) and pErk1/2 while the levels of stress-activated protein kinases (Sapk)/Jun amino-terminal kinases (Jnk) did not change (Figure 8C and Figure S12). To understand further the effect of B18L, we examined levels of Bak and Bid in B18L treated cells. Addition of B18L to cells increased the levels of Bak, Bid and tBid (Figure 8D and Figure S12). Furthermore, we examined the levels of caspase 3 (Cas3) and the caspase substrate, poly (ADP-ribose) polymerase-1 (PARP-1). We found that B18L induced an increase in Cas3 cleavage and promoted the cleavage of PARP-1 to the characteristic apoptotic fragment (Figure 8E and Figure S12), suggesting potential DNA fragmentation. From these results, we conclude that B18L-mediated negative regulation of the Src/Erk1/2, are likely to be involved in the expression and activities of BH3-only member of Bcl2 family of apoptotic proteins and subsequent killing of MCF7-G11-TR5 cells. It is therefore tempting to state that B18L has polyfunctional anti-cancer property triggered by a cascade of events that involves both cell membrane and intracellular targets (Figure 8F), the result of which is a multifaceted cell killing approach (Figure 8G).

## 3. Discussion

The decision for a cancer cell to live or die depends on a variety of integrated apoptotic and survival signals induced intrinsically or by therapeutic agents. In some cases, a common pathway or factor integrates the apoptotic and survival signals or arbitrate between them. One such factor is BST-2, which is an IFN-inducible type II transmembrane protein that is overexpressed in various cancers [30,31], including breast cancer [2,9,10].

We have previously described the anti-adhesion activities of the BST-2-based peptides B49/B49Mod1 series [2] and the first generation analog B18 [11]. While B18 is effective in inhibiting cancer cell adhesion, the peptide has minor cytotoxic activity against cancer cells [11]. In this manuscript, we report the structure-activity-directed studies of B18 peptide that led to the development of a series of five B18-derived peptidomimetics—B18K, B18KL, B18KA, B18L, B18I. Replacement of Phe (F) with Leu (L) but not Ile (I) at position 2 and Lys (K) with Leu and not Ile at position 6 increased cytotoxic activity compared to all other substitutions (Table 1, Table 2 and Table 3). Hence, B18L is the culmination of our efforts into the design of novel and selective anti-cancer peptidomimetic, coined from B18, a derivative of B49/B49Mod1 [2].

Based on initial cytotoxic screens, B18L was selected for further characterization. B18L is a cationic compound with random/flexible structure in aqueous solution and 42% helical character when in 1/9 peptide/POPC ratio. With the use of MD simulations, a possible mechanism of action of B18L emerged showing that B18L binds BST-2 (Figure 2) and a model POPC/POPS membrane (a mimic of cancer cell membrane, Figure 5). The simulations show that binding of B18L to BST-2 occurs early and that the interaction is stable and involves the formation of hydrogen bonds. The MD simulation on B18L’s binding to BST-2 was validated with wet-bench studies indicative of B18L•BST-2 interaction (Figure 2I–K). Aside from the interaction between B18L and BST-2, we used MD simulations to study the dynamic changes of B18L in model POPC/POPS membrane. B18L stably bound POPC/POPS membrane for 1000 ns simulation time during which time, both compound helicity and RMSD analysis show that B18L is stable on the membrane (Figure 5). Additionally, atomistic level observation indicates that the presence of B18L leads to the formation of water channel with stable pore structure whose size depends on the number of simulated monomers (Figure 5). The ability to form pores/water channel is a feature of membrane active peptides and was observed in the parental B18 peptide [11]. Within the limits of simulation, we observed various binding interactions that includes electrostatic and intramolecular bindings. These bindings represented by hydrogen bonds was much greater with six monomer B18L bundle compared to four monomer bundle. Such hydrogen bonds may essentially encourage intermolecular interactions between B18L molecules or B18L and other surface molecules, such as BST-2 or integrins.

At the cellular level, B18L induces rapid killing of tamoxifen resistant MCF7-G11-TR5 at an IC_50_ (3.8 ± 0.3 μM) lower than applicable for non-cancerous MCF-10a (17.2 ± 6.4 μM), PBMCs (39.8 ± 3.8 μM) and EC_50_ of 21.2 ± 3.2 μM for erythrocytes. While the higher EC_50_ of B18L for erythrocytes may stem from the hydrophobic nature of their membranes compared to cancer cells, the higher IC_50_ for PBMCs may be the result of low BST-2 protein on the surface of PBMCs compared to tumor cells [32]. Whether or not the amount of BST-2 expressed in a cell regulates interaction with B18L and the resultant cytotoxic effect on cancer cells remains to be determined, although MCF7-G11-TR5 have higher BST-2 and are more susceptible to B18L compared to parental MCF7 cells (Figure 4). While the mechanisms of action of B18L is unclear, data presented herein indicate that the anti-cancer activity of B18L stems from a direct plasma membrane destruction and release of cytoplasmic contents. We observed that B18L induces bleb formation and membrane deformation in the tamoxifen resistant MCF7-G11-TR5 cells. The blebs appear within ~10 s of addition of B18L. Impaired membrane integrity allows internalization of PI (a red-fluorescent intercalating agent that permeates compromised cell membrane to stain DNA) and release of cytoplasmic LDH. While the function of B18L-induced membrane blebs is unknown, blebbing has been proposed to promote autophagic apoptosis [33]. In general, the B18L-induced cell membrane deformations is a desirable property of anti-cancer peptides (ACPs), which has emerged as potential alternative anti-cancer therapeutics.

The structural properties of B18L suggest a cationic amphiphilic compound. These features of B18L are similar to current ACPs that are i) mostly derived from anti-microbial peptides, ii) amphiphilic low molecular weight peptides (5 to 30 amino acids), iii) consist of net positive charge (+2 to +9) and iv) are either α-helical, β-sheet or extended/random coil structures [34]. The net positive charge facilitates binding of ACPs on cancer cell membranes that consist of net negative charge on their surfaces due to the presence of anionic molecules like heparan sulfate, sialic acid and phosphatidylserine (PS). It is well known that PS is externalized in cancer cells compare to normal cells [35]. Indeed, anionic components on the outer leaflet of cancer cell membranes, such as PS is expected to enhance binding of B18L to the cell membrane. In our study, MD simulation predicts that the presence of B18L on the membrane results in aggregation of PS around the compound (Figure 5). This observation was confirmed in functional assays where addition of B18L to MCF7-G11-TR5 cells results in time-dependent increase in Annexin V intensity, an indication of increased PS externalization. MCF7-G11-TR5 cells with externalized PS also exhibit nuclear PI stain, which is consistent with cell death (Figure 7D). Indeed, Annexin V/PI stain has been used for detection of cancer cell apoptosis with other anti-cancer peptides [36,37,38].

The Annexin V•PS complex occurred as early as 30 s after the addition of B18L. Since the Annexin V•PS occurred very early, it is possible that B18L induces the externalization of PS which may serve as “eat me” signals at the cell surface [39]. Indeed, surface expressed PS has been used as a target for detecting apoptosis using Annexin A5 antibody both in vitro [40], in animal models [41] and in patients [42,43]. PS is expressed at the cell surface very early following the onset of execution of apoptosis, which accordingly correlates with increased activities of the pro-apoptotic BH3-only member of Bcl2 family of apoptotic proteins—Bak and Bid, increased levels of Cas3 cleavage products p19, p17 and p12, as well as the cleavage of PARP-1 (Figure 8E).

The dynamics of plasma membrane in the presence of B18L includes membrane blebbing, deformation and rupture, uptake of PI and externalization of PS. Membrane rupture is a good indicator of oncotic death, which we studied with LDH release into the medium [44]. All of these phenomena may occur downstream of Cas3 activation in cells undergoing apoptosis. Whether or not PS externalization and membrane blebbing are regulated together or independently is yet to be determined but it is noteworthy that Annexin V is present on B18L-induced membrane blebs (Figure 7E).

While the precise mechanisms by which B18L mediates the killing of cancer cells is unknown, several lines of evidence indicate that B18L induces membranolytic effects, negatively regulates Src•Erk1/2 signaling and activates the extrinsic and mitochondrial apoptotic pathways, as evidenced by its cleavage of Cas3, Bid and PARP (Figure 8). Although it is premature to estimate the therapeutic index (TI) of B18L at this developmental stage, comparison of the IC_50_ of MCF-10a estimates preliminary TI of B18L as 4.5. This result indicates that while B18L exhibits acceptable selectivity, additional improvements of the compound may facilitate creating analogs with balanced DRBC-specific safety vs. efficacy profile.

Thus, future development of B18L will focus on assessing Absorption, Distribution, Metabolism and Excretion (ADME) properties of B18L and determining in vivo efficacy, tissue distribution and tumor residence time in relevant mouse models to determine if in vitro activity of B18L translates to in vivo efficacy. We will also determine safety profile of B18L, including long-term interval drug effect against different cell lines, especially MCF7-G11-TR5, as well as evaluating the interaction between B18L and BST-2.

## 4. Materials and Methods

### 4.1. Ethics

Blood samples were collected from study participants who at the time of collection, had no symptoms of infection and reported not using illicit substances. Studies were conducted according to University regulations approved by Stony Brook University Institutional Review Boards (IRB # 201900507).

### 4.2. Chemical Reagents

Roswell Park Memorial Institute (RPMI) 1640 media, Dulbecco’s Modified Eagle Medium (DMEM) media, penicillin-streptomycin, Amphotericin B, l-gutamine and 1x Dulbecco’s phosphate buffered saline (DPBS) were obtained from Corning, Thermofisher, Grand Island, NY, USA. 4-(2-hydroxyethyl)-1-piperazineethanesulfonic acid (HEPES) was obtained from Fisher Biotech, Fair Lawn, NJ, USA. Fetal bovine serum (FBS) was obtained from Atlanta Biologicals, Flowery Branch, GA, USA. Ficoll-Paque PLUS was obtained from Amersham Biosciences, Uppsala, Sweden. Tris and NaCl were obtained from Thermos Scientific, Fair Lawn, NJ, USA. Albumin, Bovine Fraction V (BSA) and Triton X-100 were obtained from Research Products International, Mount Prospect, IL, USA. Cell viability imaging kit (blue/green) was obtained from Thermofisher, Grand Island, NY, USA. eGFP Annexin V and PI Apoptosis Kit were obtained from GeneCopoeia, Rockville, MD, USA. 3-(4,5-dimethylthiazol-2-yl)-2,5-diphenyl tetrazolium bromide (MTT) reagent was purchased from Sigma-Aldrich, St. Louis, MO, USA. CellTiter-Glo reagent was obtained from Promega, Madison, WI, USA. Acetic acid was obtained from Macron Fine Chemicals, Radnor, PA, USA. 1-palmitoyl-2-oleoyl-glycero-3-phosphocholine (POPC) was obtained from Avanti polar lipids, Alabaster, AL, USA. Primary antibodies pErk1/2, Erk1/2, pSrc, Src, cCas3, Cas3, Itg α5, Itg β1, pSapk/Jnk, Sapk/Jnk, pBim, Bim, Bak and Bid were obtained from Cell Signaling Technology, Danvers, MA, USA. Primary antibody PARP-1 was obtained from Santa Cruz Technology, Santa Cruz, CA, USA. Primary antibody β-actin was obtained from Proteintech, Chicago, IL, USA.

### 4.3. Cell Lines

MDA-MB-468, MDA-MB-231, ZR-75-1, T47D, MCF7, MCF7-G11-TR1 and MCF7-G11-TR5 were obtained from the Physical Sciences-Oncology Network (PS-ON) Bioresource Core Facility (PBCF) at American Type Culture Collection (ATCC, Manassas, VA, USA). MCF7-G11-TR1 and MCF7-G11-TR5 are MCF7 derivatives that are Tamoxifen-resistant at 1 µM and 5 μM respectively. We do not have the point-by-point protocol for generating these cells. SKBR3 was a kind gift from Dr. Hyungjin Kim of Stony Brook University. Cells were maintained according to supplier’s guidelines. PBMCs were isolated from fresh human leukapheresis products by Ficoll-Paque PLUS and maintained in complete RPMI 1640 media.

### 4.4. Peptide Synthesis

The peptides were synthesized by ChinaPeptides (Shanghai, China) with a purity of >95% with Trifluoroacetic acid (TFA) removal. The peptides were resuspended with 0.02% acetic acid in ultra-pure water into 5 mg/mL aliquots and stored at −20 °C until used for the other experiments.

### 4.5. CD Analysis

POPC liposome was prepared as described previously [45]. Briefly, POPC dissolved in chloroform was allowed to dry leaving a thin lipid film in a glass vial. The film was re-hydrated with Tris buffer (0.5 mM, pH = 7.4) to generate multilamellar vesicles (LUVs) at room temperature. The LUVs suspension was extruded through 1 µm polycarbonate filters five times to obtain the small unilamellar vesicles (SUVs). CD analysis of the compounds with or without POPC SUVs was conducted through a Jasco J-715 spectrophotometer (Jasco Analytical Instruments, Easton, MD, USA). Each sample was measured three times with a 1 mm path length quartz cuvette and the full spectrum from 190 to 260 nm was collected at 0.1 nm resolution with a scan rate of 100 nm/min. The data was analyzed by the CAPITO web tool [46].

### 4.6. Viability Assay

Cells were plated at 5000 cells/well (cancer cells) or 10,000 cells/well (PBMCs) in a 96-well plate and allowed to attach for 24 h. Cells were then treated with various concentrations of compounds for the times indicated in the different figures. At each time point, the viability of cells was measured using MTT assay. In brief, each well was treated with 20 µL of MTT reagent (5 mg/mL in 1x DPBS) and incubated at 37 °C for 3 h. After 3 h, 100 µL of MTT solvent (90% Isopropanol, 10% Triton X-and 0.4% 1M HCl) was added to each well and the plate was covered from light while shaking for 20 min. The absorbance of solubilized MTT reagent was measured using spectrophotometer at 590 nm. In some experiments with PBMCs, the ATP assay was used to assess cell viability by following manufacture’s instruction. Briefly, 10,000 cells/well was plated in 96-well plate and treated with various concentrations of B18L. Cells were then incubated at 37 °C for the indicated time points on the figure and then treated with equivalent volume of CellTiter-Glo reagent. The plate was rocked for 10 min and luminescence intensity was read using spectrophotometer.

### 4.7. Hemolysis Assay

The hemolysis assay was done by following Duvall’s method [47]. Under pre-approval from IRB, 10 mL of blood from each of three anonymous human donors were collected and drawn directly into K2-EDTA-coated Vacutainer tubes (Becton Dickinson, Thermofisher, Grand Island, NY, USA) to prevent coagulation. The blood was then centrifuged at 500× *g* for 5 min to remove plasma. The erythrocytes were washed with equivalent volume of 150 mM NaCl solution and 1x DPBS twice respectively by spinning at 500× *g* for 5 min. The erythrocytes were then diluted with 1:50 with 1x DPBS and 190 μL diluted cells were added to the 96-well round bottom plate after 10 μL of B18L compounds were added to the plates with the corresponding concentrations. 20% Triton X-100 was used as positive control. After 45 min or 24 h treatments, the plates were spun at 500× *g* for 5 min. 100 μL supernatant was transferred to a 96-well flat bottom and absorbance was measured with a plate reader at 490 nm.

### 4.8. Real-Time Quantitative PCR (RT-qPCR)

Equivalent amount of RNA from MCF7 and MCF7-G11-TR5 cells were used for cDNA synthesis using the High Capacity cDNA Reverse Transcription Kit (Applied Biosystems, Thermofisher, Grand Island, NY, USA). The cDNA was then applied for RT-qPCR assay. The thermal cycler program and expression calculation was setup in a 7500 FAST real-time PCR system (Applied Biosystems, Thermofisher, Grand Island, NY, USA) and the fold change in gene expression was calculated using the standard ∆∆CT method. The primers used for human BST-2 was forward: 5′-AAGTACTACCCCAGCTCCCA and reverse: 5′-TGTTCAAGCGAAAAGCCGAG. GAPDH was used as loading control and the primers were reported previously [48].

### 4.9. Western Blot

Equivalent numbers of MCF7-G11-TR5 cells were plated and allowed to attach 24 h before exposure to appropriate treatments. At each time point, cells were lysed using RIPA lysis buffer and total protein amount was quantified using Bradford assay. Equal amounts of protein samples at each time point were loaded into 4–20% Mini-PROTEAN TGX precast gels (Bio-Rad, Hercules, CA, USA) and resolved by SDS-PAGE at a constant 100 V and then blotted onto PVDF membranes (Bio-Rad, Hercules, CA, USA). Membranes were blocked with 5% BSA dissolved in 1x TBST (50 mM Tris, 150 mM NaCl and 0.1% Tween, pH 7.6) buffer for 1 h at room temperature on slow shaker. The membranes were incubated at 4 °C overnight with primary antibody. β-actin was probed after stripping the membrane and used as loading control. After 10 min washes with 1x TBST, membranes were incubated with appropriate IRDye secondary antibodies (Licor, Lincoln, NE, USA) at room temperature on slow shaker for 1.5 h. The membranes were then washed in 1x TBST for 5 min prior to band detection using the Odyssey Infrared Imaging System (Licor, Lincoln, NE, USA).

### 4.10. Molecular Dynamics (MD) Simulation

The simulation system setup has been described previously by us [11] and others [17,49,50]. Briefly, for B18L•BST-2 system, two B18L chains were initially placed 5 nm away from the center of BST-2. For the B18L•membrane system, four B18L chains were initially placed on the surface of cell membrane or in the center of cell membrane, six B18L chains were inserted in the transmembrane at t = 0. The systems were then constructed by the CHARMM-GUI web tool [51,52]. The systems were filled with 0.15 M KCl to a final neutral charge. The simulation environment was set as periodic boundary conditions (PBC) with the Particle mesh Ewald (PME) method [53]. CHARMM36 force field [52,54] and GROMACS 5.1.1 [55] software were employed in all simulations. The trajectories were analyzed using VMD 1.9.3 [56] and GROMACS.

### 4.11. Analysis of Cell Morphology by Live-Imaging Microscopy

Prior to treatment, 10,000 MCF7-G11-TR5 cells were plated in a 96 well plate 18 h. Following B18L treatment, 10x live-kinetic Brightfield images were captured at 30 s intervals for 45 min using the Lionheart FX automated microscope’s experiment mode feature (Biotek, Winooski, VT, USA). Before treatment with B18L, images were taken as time 0. For live/dead cell stain, cells were pre-stained with Cell viability imaging kit (blue/green) for 1 h prior to treatment with peptide. Cells were treated with peptides (IC_50_ concentration) and 10x live kinetic images were taken at 5-min intervals for 45 min. The area of the cells, membrane blebs and dead cell intensity were quantified using ImageJ 1.52a [57].

### 4.12. Annexin V and PI Staining Assay

Prior to treatment, 10,000 MCF7-G11-TR5 cells were seeded in a 96 well plate 24 h with B18L at respective time points (0, 0.5 min, 5 min, 10 min, 15 min, 45 min). Following treatment, cells were incubated with eGFP Annexin V and PI in 1x Annexin-binding buffer at room temperature for 30 min in the dark. Cells were washed with 1x Annexin-binding buffer and fluorescence images were manually obtained at 10x using the Lionheart FX automated microscope (Biotek, Winooski, VT, USA). Five 10x fields of view were analyzed per time point using Biotek’s Gen 5 software and raw data plotted using GraphPad Prism 8 (La Jolla, CA, USA).

### 4.13. UV Spectroscopy Assay

The absorbance at 230 nm was measured to estimate compound concentrations since peptide exhibit a strong absorption at 230 nm [58,59]. B18L (MW = 1.683 kDa), the rBST-2 (MW = 5.832 kDa) and the mixture of B18L and rBST-2 (B18L•BST-2) were titrated with UV spectroscopy at 230 nm to obtain the standard curve. 2 mg/mL of B18L and 1 mg/mL of rBST-2 were preincubated at 37 °C for 45 min. 200 μL mixture was then subjected to the ultracentrifuge tube with 3000 NMWL filter and centrifuged at 13,000 rpm for 1 h. Absorbance of the compounds i) before spin designated as input, ii) compounds left on the apical side of the membrane designated as retained and iii) compounds in the basal side of the tube designated as the flow through were measured with UV spectroscopy. The standard curve was used to extrapolate protein concentrations from the absorbance measurements.

### 4.14. Statistical Analysis

GraphPad Prism was used to plot all the graphs and to determine the statistical significance in this study. For a two-group comparison, unpaired t-test with Welch’s correction was used to determine the differences between the groups. For a multiple-group comparison, ordinary one-way ANOVA test with Dunnett’s correction was used to determine the differences between the treated groups as compared to vehicle treatment. * *p* < 0.05, ** *p* < 0.01, *** *p* < 0.005, **** *p* < 0.001 and ns = nonsignificant.

## 5. Conclusions

Overall, the results presented in this manuscript suggest that B18L may induce cell death through processes involving membrane surface blebs, loss of membrane integrity, PI internalization, LDH release and PS externalization, coupled with coordinated regulation of Src•Erk1/2 signaling and mitochondrial events. In addition to direct induction of cell death, cancer cells may take up B18L through binding to PS allowing invagination and encapsulation of the compound. In this case, the membrane disruptive properties of B18L may help the insertion of current chemotherapeutic agents, which by themselves may be less likely to perturb the membrane or enter cancer cells.

## 6. Patents

Lyu Y & Okeoma CM. Compositions and Methods for Inhibition of BST-2. 2020. Patent pending—050-9025. Stony Brook University, Provisional Application Serial No. 62/985,086.

## Figures and Tables

**Table 1:** B18 and B18-derived Peptidomimetics.

ID	Sequence	# of Residues	Charge	H ^1^ (kcal/mol)	μH ^2^ (kcal/mol)	MW ^3^ (kDa)
B18	GFQDVEAQAATCNHTVMA	18	–2	0.358	0.222	1.893
B18K	GFQKVKAQAATCNHTVMA	18	2	0.326	0.224	1.905
B18KL	GFQKVKAQALTCLHTVMA	18	2	0.531	0.315	1.946
B18KA	GFQKAKAKALACLAKALA	18	4	0.357	0.375	1.803
B18L	GLGKALAKALACLAKALA	18	3	0.513	0.523	1.683
B18I	GIGKAIAKAIACIAKAIA	18	3	0.541	0.547	1.683

^1^ H: Hydrophobicity, ^2^ µH: Hydrophobic moment, ^3^ MW: Molecular weight. Gray shading represents drug lead.

**Table 2:** Initial Screening Data on breast cancer cells for 24 h.

Cell Lines	Subtype				IC_50_ (µM)				
		ER	PR	HER2	B18K	B18KL	B18KA	B18L	B18I
MDA-MB-468	Basal A	-	-	-	*>131.0	>128.5	135.6 ± 9.3	12.4 ± 1.4	16.6 ± 1.5
MDA-MB-231	Claudin-Low	-	-	-	>131.0	>128.5	83.7 ± 20.1	7.2 ± 0.8	18.6 ± 2.4
ZR-75-1	Luminal A/B	+	+/-	-	>131.0	>128.5	58.9 ± 2.0	5.2 ± 1.0	10.1 ± 1.8
T47D	Luminal A	+	+/-	-	>131.0	>128.5	78.5 ± 20.9	17.5 ± 6.5	>148.5
SKBR3	HER2	-	-	+	>131.0	>128.5	43.5 ± 11.5	7.2 ± 1.0	18.1 ± 4.7

* > means IC_50_ is higher than the highest tested compound concentration.

**Table 3:** Activity of B18L against DSBC and DRBC cell lines for 24 h.

Cell lines	Subtype				IC_50_ (µM)				
		ER	PR	HER2	B18K	B18KL	B18KA	B18L	B18I
MCF7	Drug-sensitive	+	+	-	*>131.0	>128.5	61.8 ± 3.9	5.9 ± 0.5	16.0 ± 1.6
MCF7-G11-TR1	Drug-resistant (1 µM)	+	+	-	>131.0	>128.5	61.8 ± 5.1	6.2 ± 0.8	10.8 ± 2.0
MCF7-G11-TR5	Drug-resistant (5 µM)	+	+	-	>131.0	>128.5	56.4 ± 5.0	3.8 ± 0.3	16.1 ± 4.9

* > means IC_50_ is higher than the highest tested compound concentration.

## Supplementary Materials

The following are available online at https://www.mdpi.com/2072-6694/12/9/2448/s1, Figure S1. Original western blot replicates for Figure 4A, Figure S2. Western blot replicates for Figure 4E, Figure S3. Original western blot images for Figures 4D and S2, Figure S4. Original western blot replicates for Figure 4E quantification and Figure S2, Figure S5. Hydrogen bond (Hbond) information of four B18L peptides at membrane surface status, Figure S6. Structural properties of four B18L peptides at transmembrane status, Figure S7. Hydrogen bond (Hbond) information of four B18L peptides at transmembrane status, Figure S8. Structural properties of six B18L peptides at transmembrane status, Figure S9. Hydrogen bond (Hbond) information of six B18L peptides at transmembrane status, Figure S10. Membranolytic effects of B18L, Figure S11. Analysis of B18L on Annexin V binding to PS, Figure S12. All western blot replicates and densitometry readings for Figure 8, panels B to E., Figure S13. Original Western blot images for Figure 8, panels B to E., Figure S14. Original western blot replicates for Figure S12 repeat2, Figure S15. Original western blot replicates for Figure S12 repeat 3, Table S1. Densitometry readings/intensity ratio of each band for Figures 4A and S1, Table S2. Densitometry readings/intensity ratio of each band for Figures 4E and S2, Table S3. Densitometry readings/intensity ratio of each band for Figure 8, panels B to E and S12.
