# Supplementary material for: Development of a Cationic Amphiphilic Helical Peptidomimetic (B18L) As A Novel Anti-Cancer Drug Lead
Source: Cancers (Basel). 2020 Aug 28;12(9):2448. doi: 10.3390/cancers12092448 (PMC7563317; doi:10.3390/cancers12092448)
Supplement: Supplementary file 1 [file cancers-12-02448-s001.pdf]

cancers

Supplementary Materials:

Development of a Cationic Amphiphilic Helical Peptidomimetic (B18L) As A Novel Anti-Cancer Drug Lead

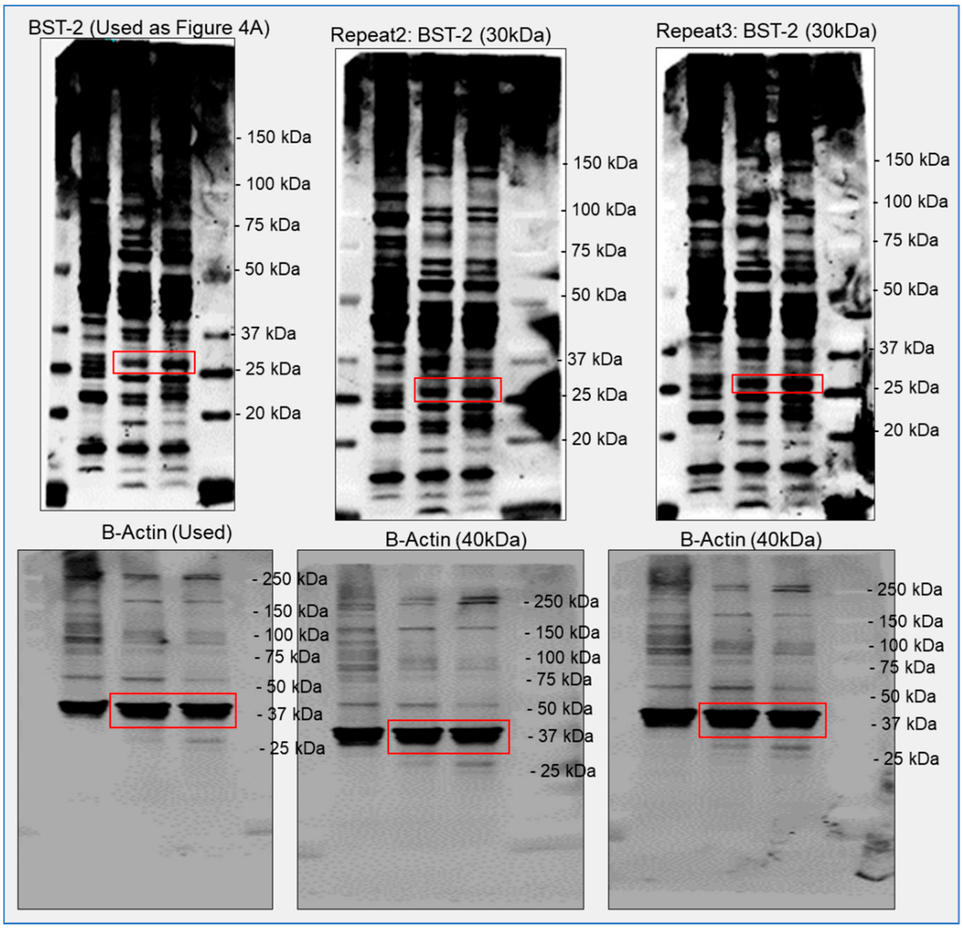

Figure S1. Original western blot replicates for Figure 4A.

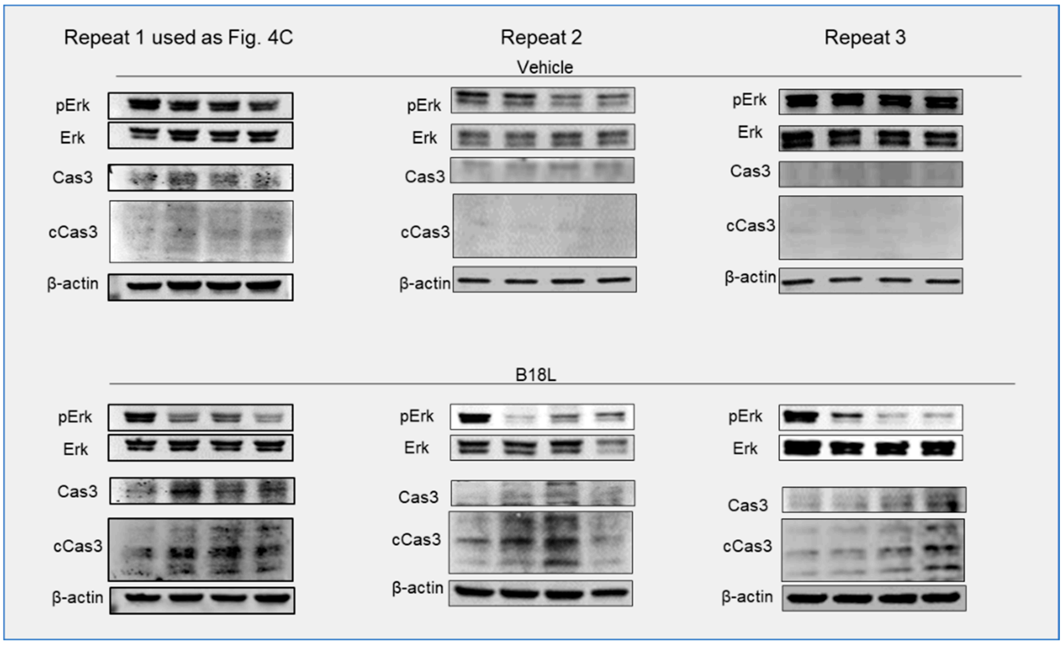

Figure S2. Western blot replicates for Figure 4E.

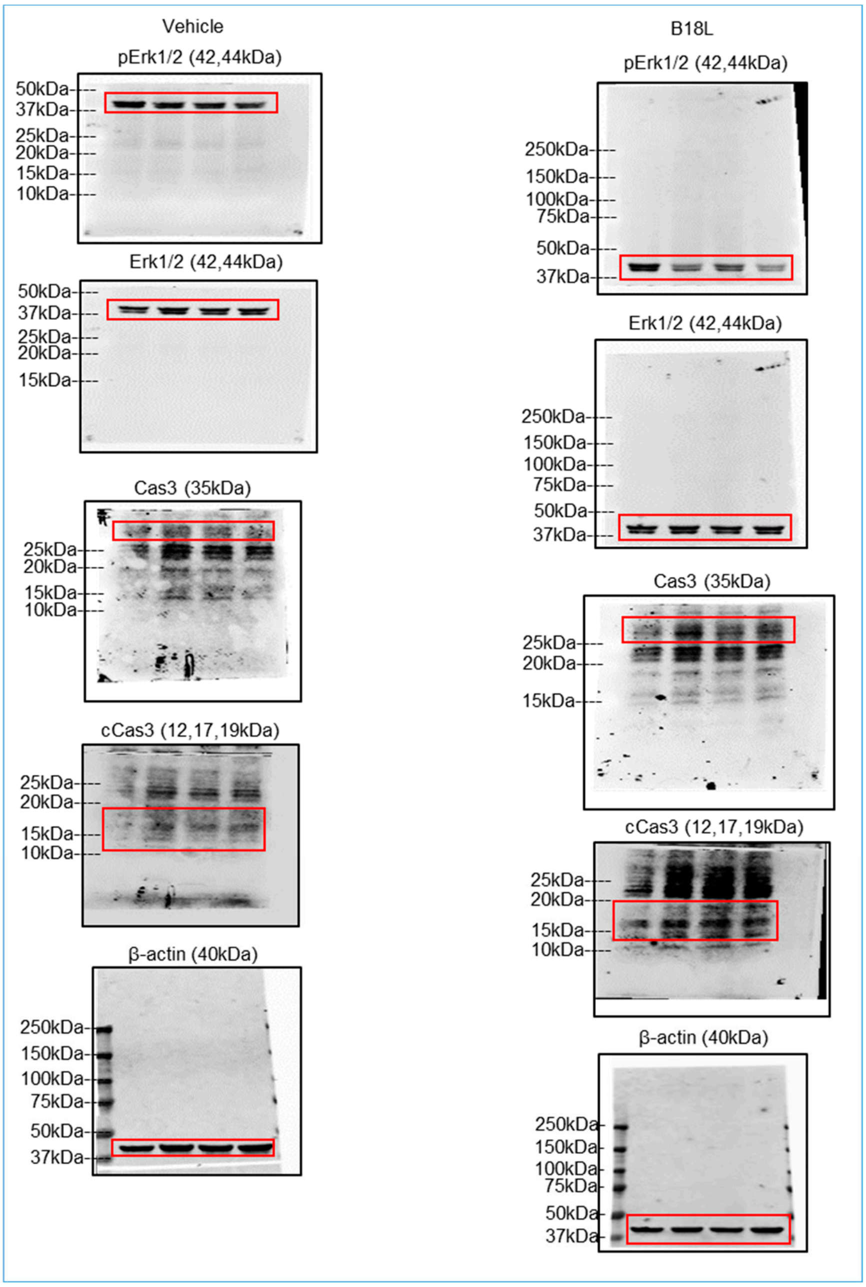

Figure S3. Original western blot images for Figure 4D and S2.

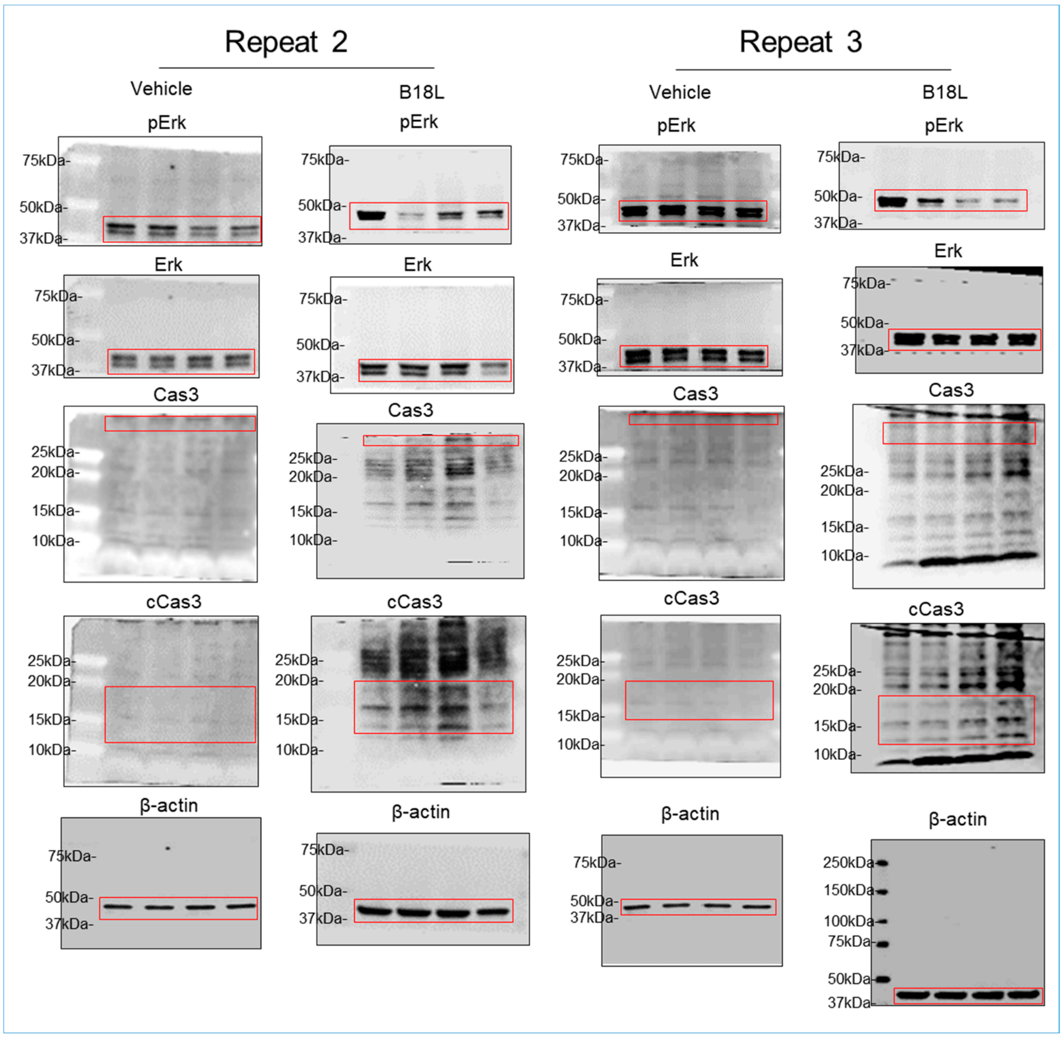

Figure S4. Original western blot replicates for Figure 4E quantification and Figure S2.

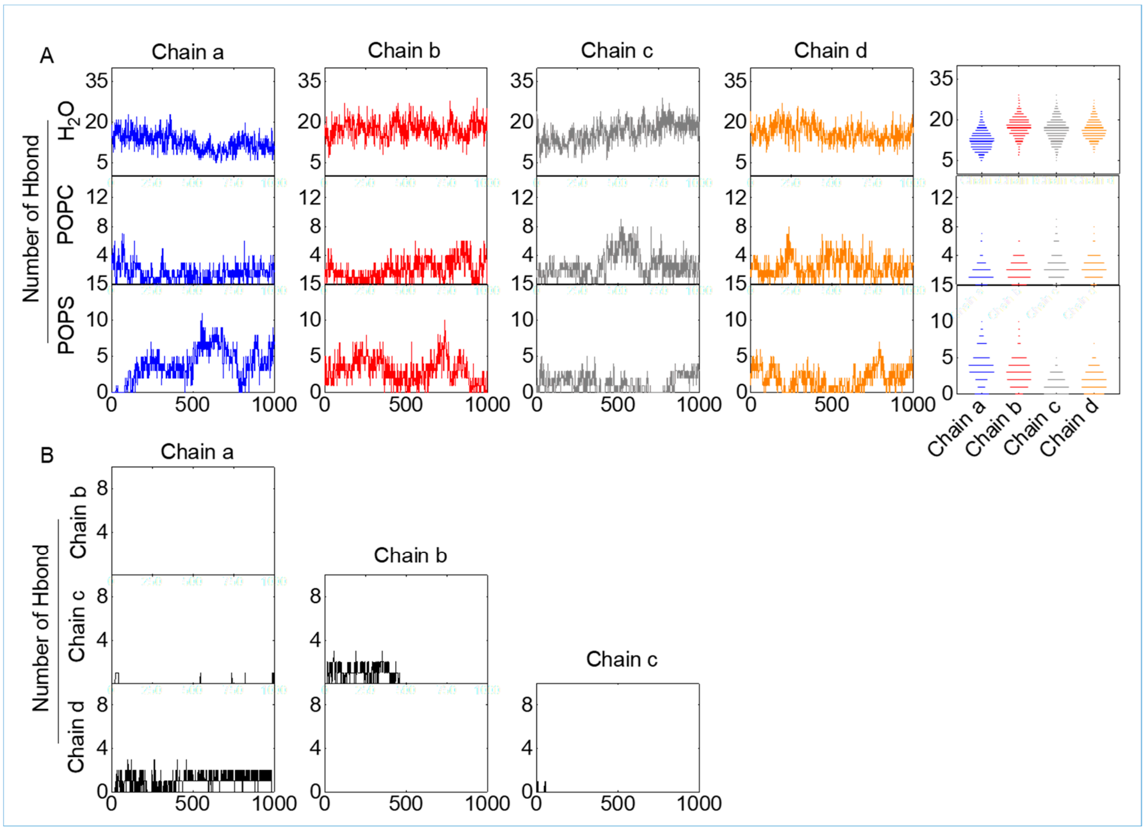

Figure S5. Hydrogen bond (Hbond) information of four B18L peptides at membrane surface status. (**A**) Hbonds between B18L chains and water, POPC and POPS. (**B**) Hbonds between B18L chains.

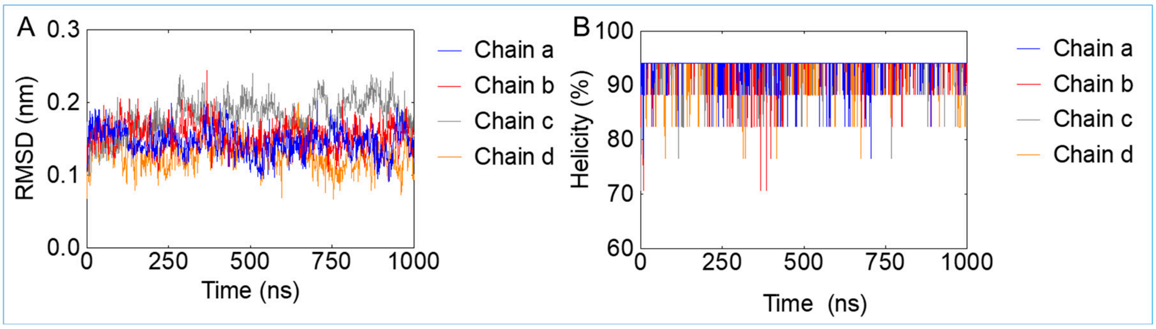

Figure S6. Structural properties of four B18L peptides at transmembrane status. (**A**) RMSD and (**B**) Helicity of each B18L chain.

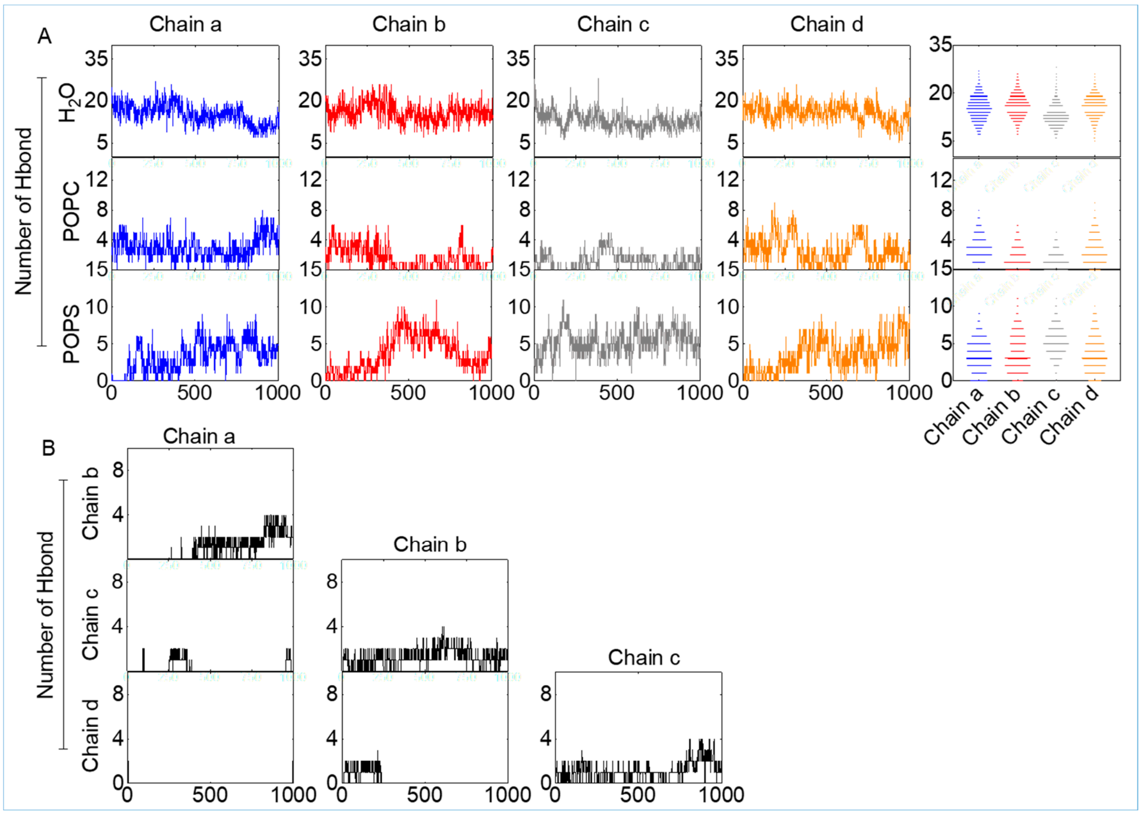

Figure S7. Hydrogen bond (Hbond) information of four B18L peptides at transmembrane status. (**A**) Hbonds between B18L chains and water, POPC and POPS. (**B**) Hbonds between B18L chains.

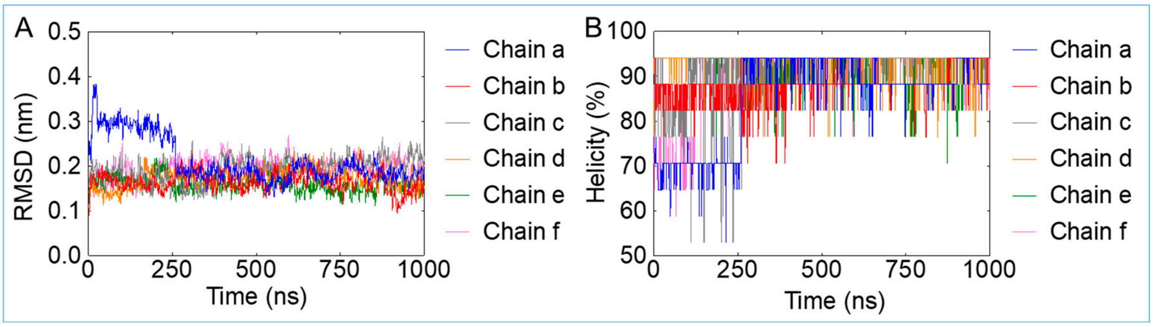

Figure S8. Structural properties of six B18L peptides at transmembrane status. (**A**) RMSD and (**B**) Helicity of each B18L chain.

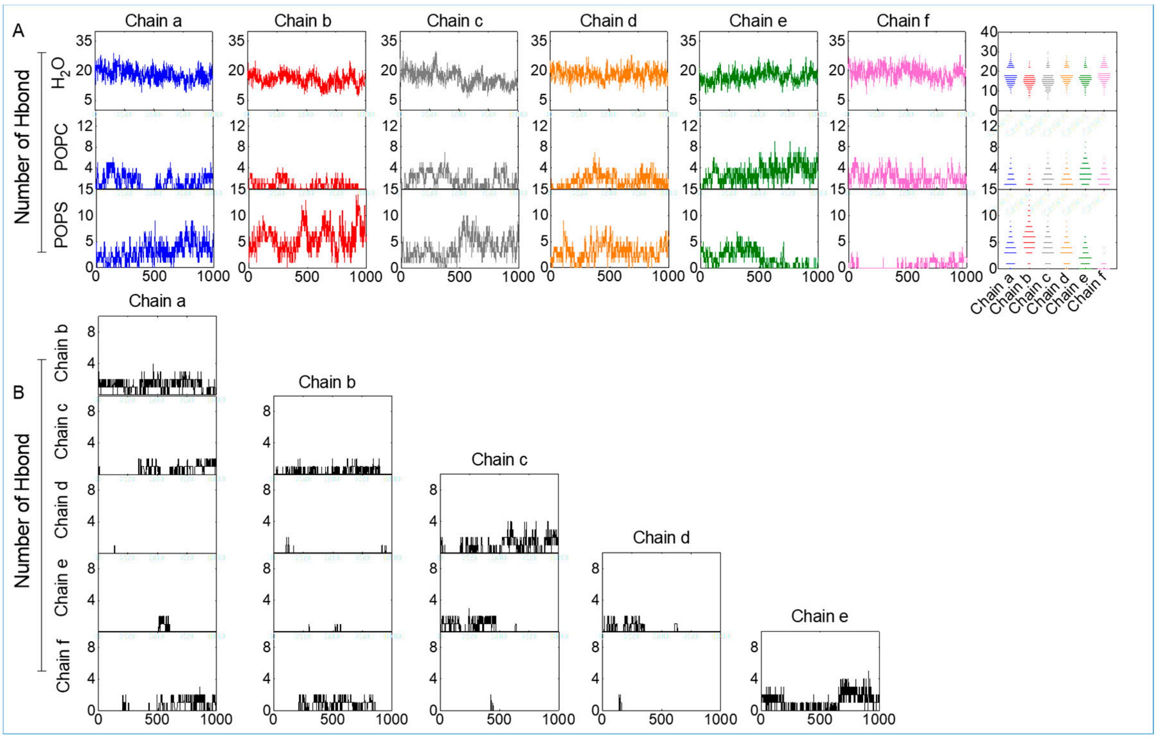

Figure S9. Hydrogen bond (Hbond) information of six B18L peptides at transmembrane status. (**A**) Hbonds between B18L chains and water, POPC and POPS. (**B**) Hbonds between B18L chains.

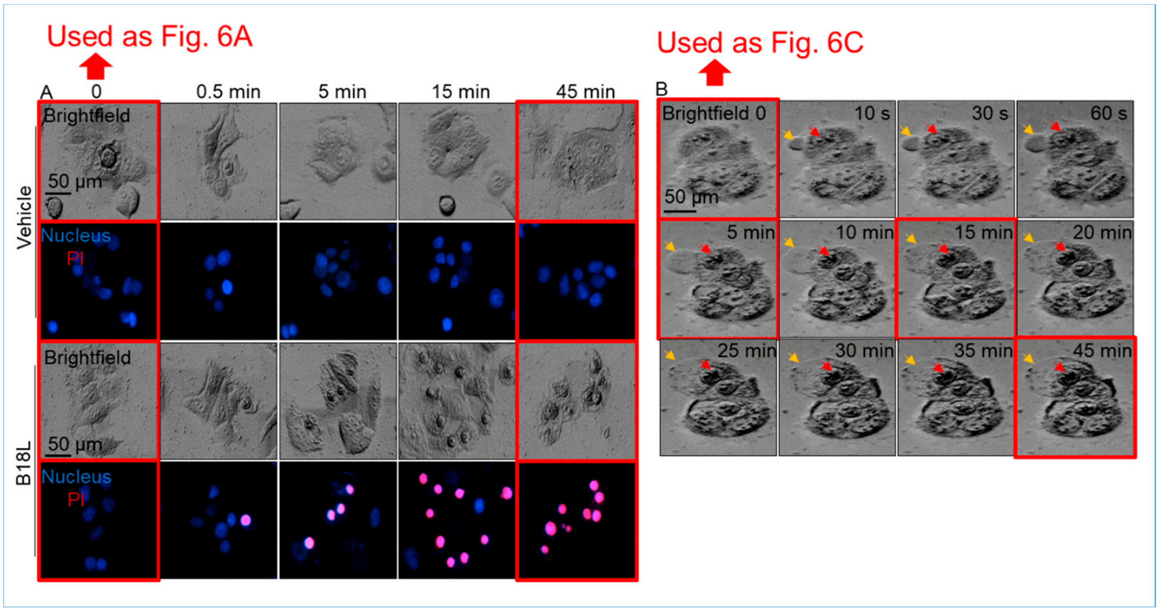

Figure S10. Membranolytic effects of B18L. (**A**) DAPI/PI staining of MCF7-G11-TR5 treated with vehicle or B18L at various time points. (**B**) Representative live cell images of membrane blebs captured at various time points.

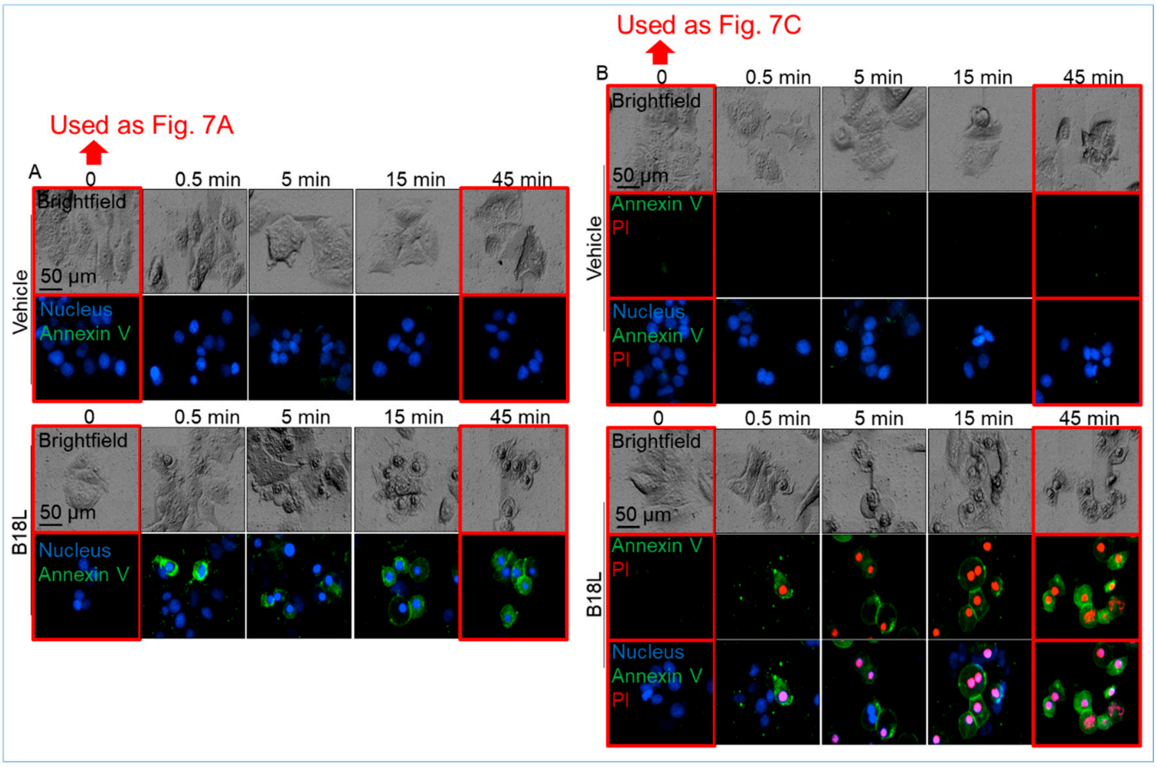

Figure S11. Analysis of B18L on Annexin V binding to PS. (**A**) Pattern of DAPI and Annexin V staining of MCF7-G11-TR5 cells following vehicle (upper) or B18L (lower) treatment at various time points. (**B**) Pattern of DAPI, PI, and Annexin V staining of MCF7-G11-TR5 cells following vehicle (upper) or B18L (lower) treatment at various time points.

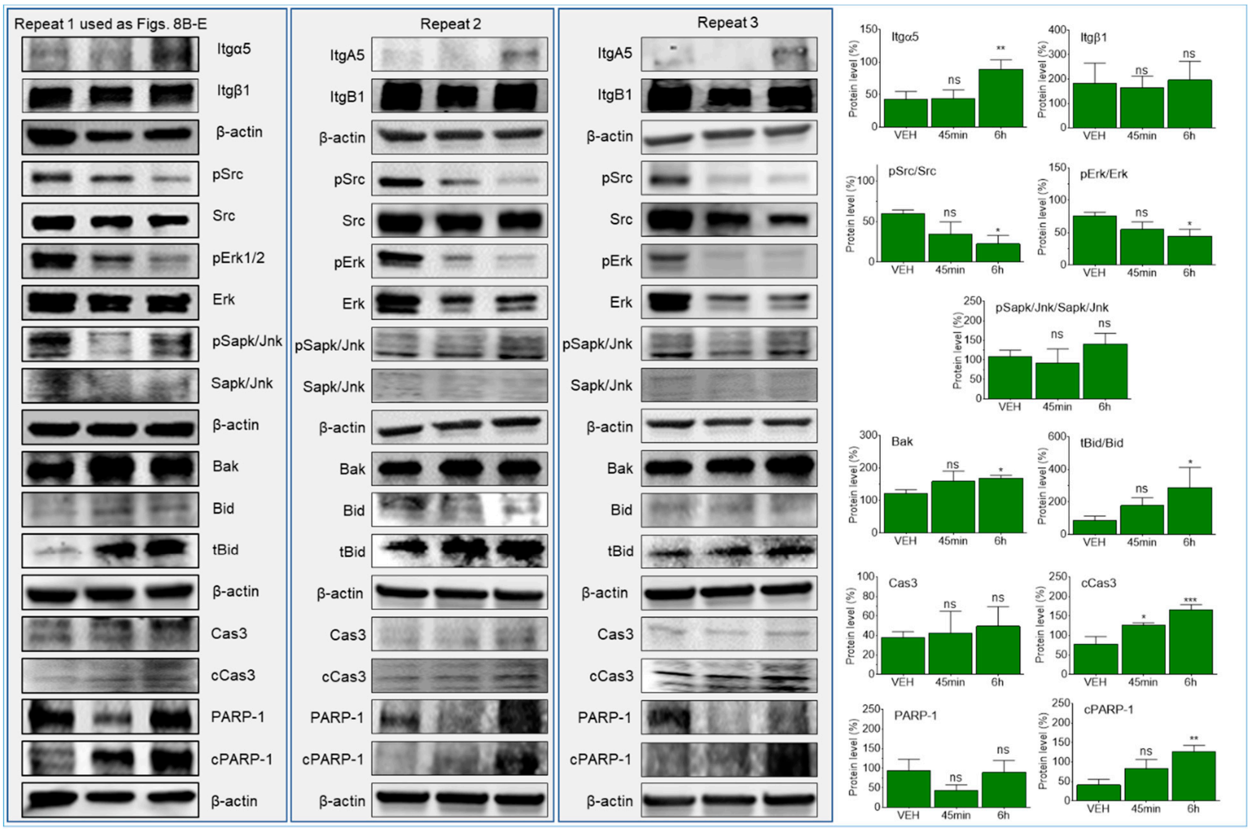

Figure S12. All western blot replicates and densitometry readings for Figure 8, panels B–E. Ordinary one-way ANOVA test (Dunnett's correction) was used to determine the differences between the treated groups as compared to vehicle treatment. *p < 0.05, **p < 0.01, ***p < 0.005 and ns = nonsignificant.

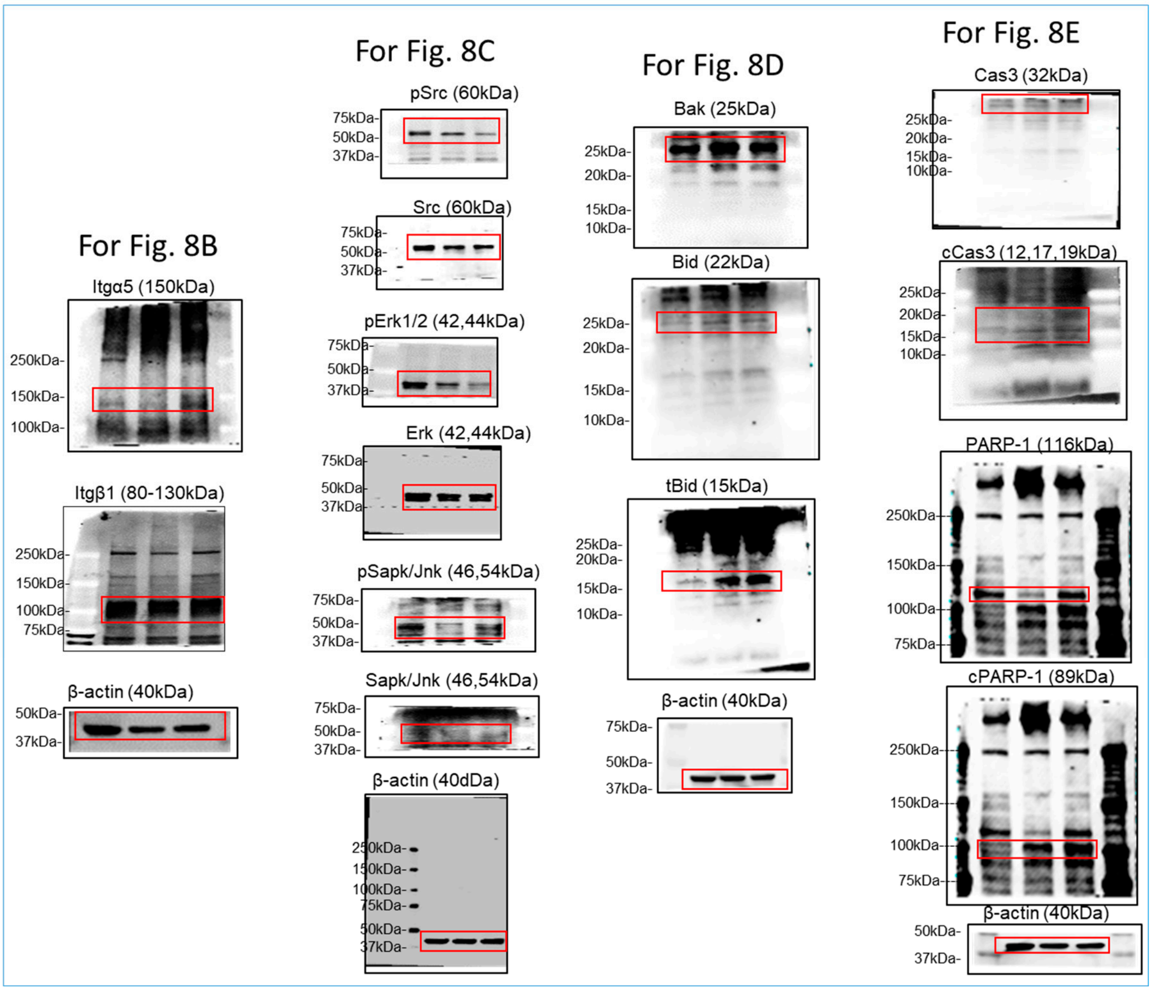

Figure S13. Original Western blot images for Figure 8 panels B to E, and Figure S12.

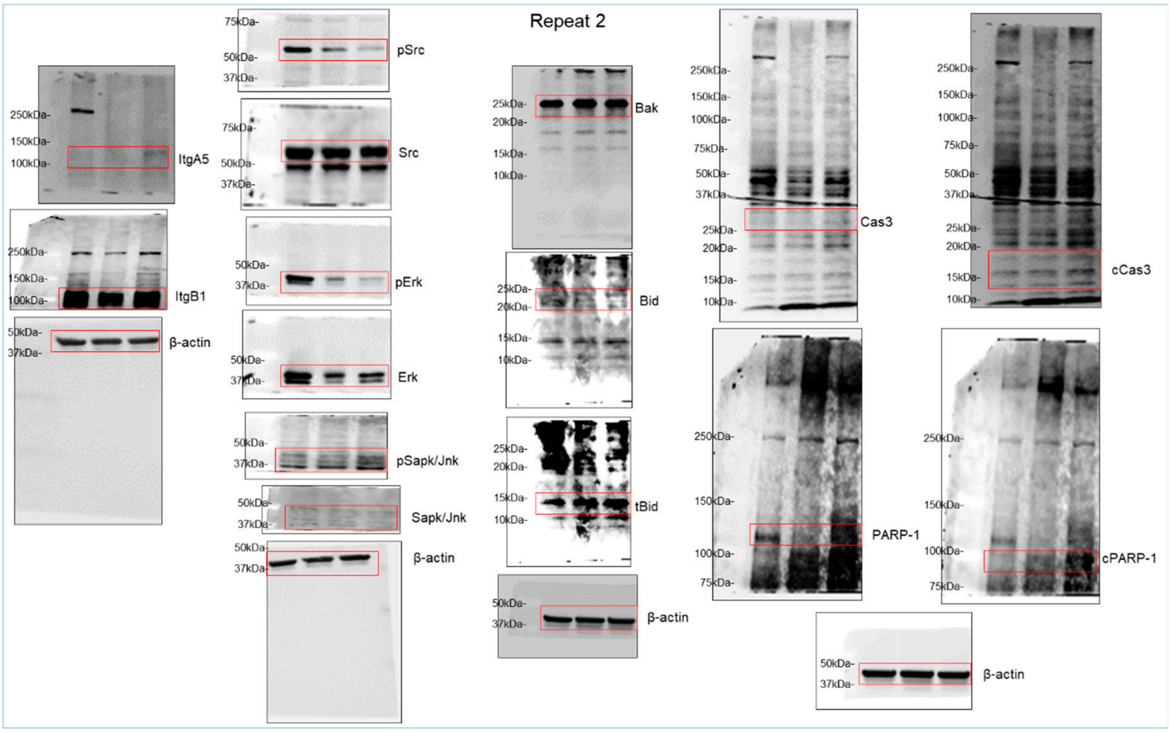

Figure S14. Original western blot replicates for Figure S12 repeat2.

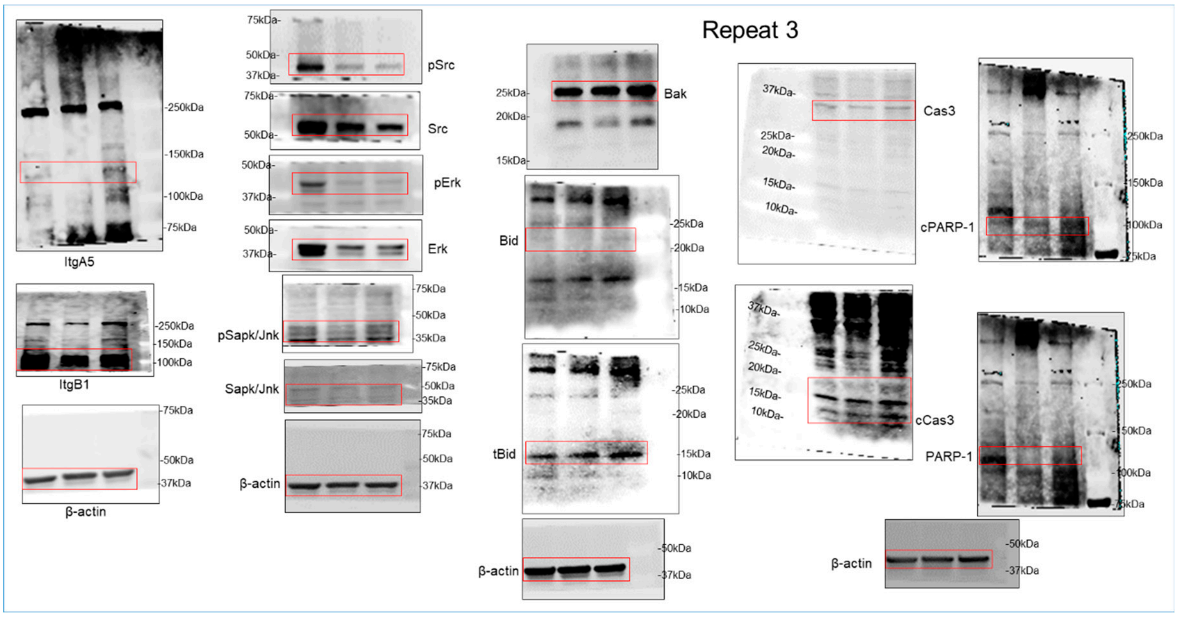

Figure S15. Original western blot replicates for Figure S12 repeat3.

Table S1. Densitometry readings/intensity ratio of each band for Figure 4A and S1.

		**BST-2**	**b-Actin**	**density/ratio**
Repeat1 (used)	MCF7	215992	275995	0.783
	MCF7-G11-TR5	277380	259682	1.068
Repeat2	MCF7	236158	300844	0.785
	MCF7-G11-TR5	307441	295723	1.040
Repeat3	MCF7	238752	319242	0.748
	MCF7-G11-TR5	307429	296922	1.035

Table S2. Densitometry readings/intensity ratio of each band for Figure 4E and S2.

	**Vehicle**	**pErk**		**Erk**		**Cas3**		**p19**		**p17**		**p12**		**b-Actin**	
		**intensity**	**ratio**	**intensity**	**ratio**	**intensity**	**ratio**	**intensity**	**ratio**	**intensity**	**ratio**	**intensity**	**ratio**	**intensity**	**ratio**
repeat1	0.17h	148438	1.43	110265	1.06	52325	0.50	37718	0.36	36585	0.35	25409	0.24	103723	1.00
	6h	130702	1.14	131248	1.15	67549	0.59	60598	0.53	63242	0.55	50709	0.44	114373	1.00
	12h	125930	1.14	121861	1.10	61394	0.55	53628	0.48	60535	0.55	41210	0.37	110627	1.00
	24h	109396	0.96	123853	1.09	48371	0.43	54945	0.48	61786	0.54	44570	0.39	113444	1.00
repeat2	0.17h	140133	1.62	137319	1.59	43279	0.50	36351	0.42	50991	0.59	38937	0.45	86310	1.00
	6h	134635	1.60	135941	1.61	46755	0.55	31226	0.37	47808	0.57	36839	0.44	84307	1.00
	12h	117763	1.30	133188	1.47	50424	0.56	33626	0.37	56303	0.62	44416	0.49	90752	1.00
	24h	114384	1.30	126355	1.43	50760	0.58	38289	0.43	58312	0.66	48007	0.54	88101	1.00
repeat3	0.17h	190983	2.26	208657	2.46	62369	0.74	48647	0.57	49107	0.58	39917	0.47	84690	1.00
	6h	199968	2.30	191895	2.21	73270	0.84	53876	0.62	49469	0.57	40768	0.47	86790	1.00
	12h	199032	2.28	189449	2.17	76524	0.88	52985	0.61	45973	0.53	41850	0.48	87178	1.00
	24h	193353	2.39	178997	2.21	69910	0.86	44404	0.55	41909	0.52	34712	0.43	80960	1.00
	B18L	pErk		Erk		Cas3		p19		p17		p12		b-Actin	
		intensity	ratio	intensity	ratio	intensity	ratio	intensity	ratio	intensity	ratio	intensity	ratio	intensity	ratio
repeat1	0.17h	125604	1.03	121518	1.00	68618	0.56	81926	0.67	142536	1.17	83055	0.68	121898	1.00
	6h	83036	0.67	135330	1.09	110318	0.89	106057	0.86	184514	1.49	127240	1.03	123694	1.00
	12h	91673	0.76	129517	1.07	92506	0.77	146939	1.22	204270	1.69	136181	1.13	120581	1.00
	24h	69659	0.55	123603	0.98	94253	0.74	154903	1.22	177965	1.41	131742	1.04	126593	1.00
repeat2	0.17h	108063	0.79	135712	1.00	41056	0.30	78166	0.57	113757	0.84	68092	0.50	136099	1.00
	6h	30866	0.23	140098	1.02	70146	0.51	141264	1.03	185860	1.36	111301	0.81	136736	1.00
	12h	55245	0.39	144206	1.03	82959	0.59	149367	1.07	200325	1.43	153016	1.09	139971	1.00
	24h	53486	0.50	94571	0.88	69568	0.65	107595	1.00	112101	1.04	78978	0.73	107755	1.00
repeat3	0.17h	148777	1.07	206190	1.49	67570	0.49	88099	0.64	93250	0.67	69572	0.50	138496	1.00
	6h	74116	0.54	152232	1.11	74496	0.54	113722	0.83	123817	0.90	94250	0.69	137527	1.00
	12h	35003	0.25	156448	1.12	106604	0.76	128887	0.92	145759	1.04	109053	0.78	139640	1.00
	24h	32757	0.24	171417	1.25	146591	1.07	166789	1.21	175070	1.27	151016	1.10	137392	1.00

Table S3. Densitometry readings/intensity ratio of each band for Figure 8 B-E, and S12.

		ItgA5		ItgB1		b-Actin	
		intensity	ratio	intensity	ratio	intensity	ratio
repeat1	VHE	237169	0.45	488071	0.93	525085	1.00
	45min	233899	0.59	446094	1.13	395771	1.00
	6h	392003	0.94	475936	1.14	417815	1.00
repeat2	VHE	89421	0.29	594071	1.92	309130	1.00
	45min	115400	0.38	541455	1.77	305807	1.00
	6h	210534	0.73	603812	2.08	289700	1.00
repeat3	VHE	126898	0.53	621274	2.60	239187	1.00
	45min	89121	0.35	523506	2.05	254798	1.00
	6h	222675	1.01	588886	2.66	221272	1.00

		pSrc		Src		pErk		Erk		pSapk/Jnk		Sapk/Jnk		b-Actin	
		intensity	ratio	intensity	ratio	intensity	ratio	intensity	ratio	intensity	ratio	intensity	ratio	intensity	ratio
repeat1	VHE	187548	0.50	294707	0.78	369262	0.98	452659	1.20	419008	1.11	424881	1.13	375801	1.00
	45min	137027	0.37	265044	0.71	253529	0.68	374045	1.00	188972	0.51	357941	0.96	372331	1.00
	6h	80320	0.21	232390	0.61	170992	0.45	380283	1.00	352574	0.92	305512	0.80	381844	1.00
repeat2	VHE	222084	0.73	364097	1.20	294991	0.97	392455	1.30	278039	0.92	271782	0.90	302650	1.00
	45min	98805	0.32	351996	1.14	99248	0.32	219221	0.71	337883	1.09	277491	0.90	309761	1.00
	6h	55136	0.17	346024	1.04	81412	0.25	240423	0.72	397517	1.20	233783	0.70	332107	1.00
repeat3	VHE	230677	1.39	423055	2.54	271582	1.63	386750	2.32	333933	2.01	262872	1.58	166511	1.00
	45min	66992	0.41	279185	1.72	140689	0.87	270404	1.67	264977	1.63	258179	1.59	162348	1.00
	6h	36927	0.23	214986	1.34	138149	0.86	251032	1.56	346816	2.16	254389	1.58	160793	1.00

		Bak		Bid		tBid		b-Actin	
		intensity	ratio	intensity	ratio	intensity	ratio	intensity	ratio
repeat1	VHE	391569	1.35	110621	0.38	79360	0.27	290464	1.00
	45min	534373	1.86	156296	0.55	260380	0.91	286681	1.00
	6h	485544	1.64	143553	0.49	285227	0.96	295906	1.00
repeat2	VHE	301322	1.16	288040	1.10	187927	0.72	260806	1.00
	45min	458456	1.65	201560	0.73	464845	1.68	277475	1.00
	6h	447721	1.66	109562	0.41	468749	1.74	269996	1.00
repeat3	VHE	339684	1.16	175138	0.60	205391	0.70	292501	1.00
	45min	376161	1.25	175599	0.58	242665	0.80	301934	1.00
	6h	501414	1.78	138473	0.49	332728	1.18	281278	1.00

		Cas3		cCas3		PARP-1		cPARP-1		b-Actin	
		intensity	ratio	intensity	ratio	intensity	ratio	intensity	ratio	intensity	ratio
repeat1	VHE	148056	0.45	220546	0.67	353090	1.06	144743	0.44	331563	1.00
	45min	183269	0.66	366008	1.32	165858	0.60	280166	1.01	277621	1.00
	6h	212645	0.71	526048	1.75	373082	1.24	427374	1.42	299993	1.00
repeat2	VHE	107837	0.36	302374	1.00	185124	0.61	79205	0.26	301911	1.00
	45min	123697	0.40	392434	1.27	108731	0.35	170129	0.55	309270	1.00
	6h	143211	0.46	464111	1.50	229364	0.74	343069	1.11	309426	1.00
repeat3	VHE	82227	0.33	170233	0.68	288788	1.15	135096	0.54	250800	1.00
	45min	60520	0.22	326966	1.18	103304	0.37	252928	0.91	276970	1.00
	6h	86250	0.32	468759	1.72	193253	0.71	346230	1.27	273066	1.00
